# Data-augmented machine learning for personalized carbohydrate-protein supplement recommendation for endurance

**DOI:** 10.1038/s41598-025-23989-7

**Published:** 2025-11-17

**Authors:** Wang Xiangyu, Wu Hao

**Affiliations:** 1 Department of Physical Education, Capital Normal University, Beijing, 100048 China; 2 School of Kinesiology and Health, Capital University of Physical Education and Sports, Beijing, 100191 China

**Keywords:** Machine learning, Personalized nutrition, Carbohydrate-protein supplement, Data augmentation, Endurance performance, Computational biology and bioinformatics, Physiology

## Abstract

Carbohydrate-protein supplementation often improves endurance performance. However, effectiveness varies significantly among individuals due to unique personal characteristics. This study aimed to develop a predictive machine learning framework for personalized supplementation, with a core methodological novelty in applying a Wasserstein Generative Adversarial Network with Gradient Penalty (WGAN-GP) to address the critical issue of data scarcity. Based on 231 rowing trials, the framework utilized 46 input features covering baseline characteristics and dietary intakes. Rowing distance was the performance outcome. The machine learning pipeline first utilized a hybrid feature selection method (correlation analysis, model-based importance, and domain knowledge). Following a comparative evaluation, WGAN-GP was utilized for data augmentation. Finally, several regression models (XGBoost, SVR, and MLP) were trained to predict rowing performance. The top-performing model was used to construct a personalized supplement recommendation framework. Feature selection identified 21 key indicators from 46 initial inputs. The XGBoost model, enhanced with WGAN-GP data augmentation, demonstrated the most robust performance, achieving a strong predictive accuracy (R² = 0.53) coupled with high stability. Body weight, explosive power, and nutritional inputs were key performance predictors. This study demonstrates that a data-augmented machine learning approach can effectively model individual responses to supplementation. The developed framework provides a data-driven pathway for creating personalized nutritional strategies to optimize athletic performance.

**Supplementary Information:**

The online version contains supplementary material available at 10.1038/s41598-025-23989-7.

## Introduction

Nutritional supplementation is a cornerstone of optimizing endurance athletic performance^1–3^. The efficacy of these supplements, particularly carbohydrate-protein combinations, is not uniform^4–6^. An athlete’s response is governed by a complex interplay of individual physiological, anthropometric, and lifestyle characteristics^7,8^. Therefore, developing a predictive framework that integrates these diverse personal indicators to determine optimal supplement dosages is crucial.

Extensive research demonstrates benefits of combined carbohydrate-protein supplements (CPS). CPS can enhance endurance performance and improve recovery markers compared to carbohydrate-only (CHO) options or placebo (PLA)^4,9^. These findings largely stem from traditional randomized controlled trials (RCTs). Such RCTs typically investigate a few fixed-dose or fixed-ratio supplement protocols, for example, the evaluation of a 4:1 CHO to protein (PRO) ratio^10^. This research paradigm seeks to determine group-average effects, aiming for a universally applicable recommendation. Consequently, resulting recommendations often embody a “one-size-fits-all” approach. While crucial for establishing general guidance, this methodology inherently masks individual variability in response to supplementation.

Current supplement guidelines are predominantly based on group-average effects^11–13^. This established methodology, however, inadequately addresses pronounced inter-individual variability in responses to supplementation^14–16^. Individuals possess diverse physiological, metabolic, anthropometric, and lifestyle profiles, leading to distinct reactions to identical supplement regimens^15,17,18^. Consequently, a universal recommendation might be optimal for some individuals but suboptimal or even detrimental for others, potentially causing adverse effects such as gastrointestinal discomfort. Treating such distinct responses as statistical noise, rather than as crucial individual signals, is a fundamental limitation of prevailing strategies. Therefore, a paradigm shift towards personalized nutrition is essential^15,18–20^. Research focus should evolve from seeking a single optimal solution for populations to creating frameworks that predict and satisfy individual athlete needs. Modern computational methods, particularly machine learning (ML), offer robust tools for this advancement. ML algorithms can identify intricate, non-linear patterns within complex, high-dimensional datasets^21,22^. By integrating numerous personal indicators, these models can forecast performance outcomes under specific supplement strategies^14^, enabling the development of truly predictive and individualized recommendations.

Therefore, the primary aim of this study was to develop and evaluate a ML framework for generating personalized CPS recommendations. While foundational studies have demonstrated the feasibility of using ML for this purpose^14^, their predictive power is often constrained by the limited sample sizes common in sports science research^23^. The central methodological contribution of this work is the systematic validation of an advanced data augmentation technique—the Wasserstein Generative Adversarial Network with Gradient Penalty (WGAN-GP)—to address this critical data scarcity problem. This generative approach is embedded within a rigorous analytical pipeline that begins with hybrid feature selection to isolate the most salient predictors. This process ensures the subsequent data augmentation is applied to a high-quality, relevant feature set, thereby enhancing the potential for improved model generalization.

The remainder of this paper is organized as follows. Section 2 provides a review of the literature on ML in sports nutrition, feature selection, and data augmentation. Section 3 details the full methodology, including data acquisition, the multi-stage ML pipeline, and the personalized recommendation framework. Section 4 presents the key experimental results, including the outcomes of feature selection and the final model performance evaluations. Finally, Sect. 5 discusses the implications of the findings, acknowledges the study’s limitations, and offers concluding remarks.

## Literature review

### ML for personalized sports nutrition

The paradigm in nutrition is shifting from population-level guidelines to personalized strategies^24,25^. ML is a primary driver of this transition. ML algorithms can model complex, non-linear relationships within high-dimensional data, reflecting the intricate interplay of factors that governs an individual’s response to nutrition^24^. This capability is essential for moving beyond group-average effects and developing truly individualized nutritional interventions^22,25,26^.

ML applications in sports science are expanding rapidly. Researchers employ predictive models to forecast athletic performance, optimize training protocols, and mitigate injury risk^27^. For instance, neural networks have been used to predict badminton shot accuracy from biomechanical and eye-tracking data^28^, and regression models can quantify performance in virtual reality training environments^29^. Specific to nutrition, Wang et al. developed a model to generate personalized CPS recommendations by integrating 45 distinct individual indicators^14^. These studies demonstrate the feasibility of using ML to translate multifaceted athlete data into actionable insights.

Despite this progress, significant methodological challenges persist. Many predictive models in sports are developed on limited datasets and feature sets, which can impair their generalizability^27^. Furthermore, AI-generated recommendations, such as for exercise prescription, often lack the necessary specificity and adaptation for high-performance contexts^30^. Critical underlying issues frequently overlooked are the systematic selection of the most salient predictive features and robust strategies to address data scarcity. Addressing these two challenges is fundamental to building reliable and effective personalized nutrition frameworks.

### Feature selection in performance prediction

Feature selection is a critical step in developing predictive models from high-dimensional sports science data. The process aims to identify the most informative subset of predictors from a larger pool of initial variables^31^. Effective feature selection mitigates the risk of model overfitting, enhances the interpretability of model outcomes, reduces computational costs, and helps overcome the “curse of dimensionality” associated with complex datasets^32^.

Feature selection techniques are broadly categorized into three families: filter, wrapper, and embedded methods. Filter methods assess feature relevance using statistical measures independent of any learning algorithm; they are computationally fast but may overlook feature interactions^32,33^. Wrapper methods evaluate feature subsets using the performance of a specific predictive model. This approach can yield higher accuracy but is computationally intensive and risks selecting features that are overfitted to the chosen model^34,35^. Embedded methods integrate the feature selection process directly into the model training phase, offering a balance between the performance of wrappers and the efficiency of filters.

To overcome the limitations of individual approaches, hybrid frameworks have become a common strategy^36,37^. These methods typically combine the computational efficiency of a filter stage with the performance-oriented evaluation of a wrapper or embedded stage^36,37^. This tiered approach can effectively remove irrelevant or redundant variables early, allowing a more sophisticated analysis on a reduced set of candidate features^36,38^. Furthermore, advanced ensemble and hybrid frameworks can formally incorporate domain expertise alongside statistical criteria, improving the stability and practical relevance of the final feature subset^31,39^.

### Data augmentation for tabular sports science data

ML applications in sports science are often constrained by limited data availability. The high costs, logistical challenges, and time-intensive nature of conducting human trials restrict sample sizes, particularly in physiological intervention studies^40^. This data scarcity can prevent the effective application of complex, data-hungry models and may impair the generalization performance of any predictive model developed^41^.

Data augmentation, a process of generating synthetic data to expand a training set, offers a viable solution to this problem^23,42^. Simpler augmentation strategies are often used as a baseline. These include Random Noise Injection, which creates new samples by adding small, random perturbations to the features of existing data points^43,44^. Another common approach is Mixup, a technique that generates virtual examples by taking linear interpolations of feature vectors and their corresponding labels from pairs of samples^45^. Such methods are computationally efficient and can be effective for basic regularization.

However, these simpler techniques have fundamental limitations. Methods based on simple interpolation or noise may fail to capture the complex, non-linear correlations inherent in biomedical data, and in some scientific applications, can even generate physically implausible data instances^40,46^. This necessitates more sophisticated approaches. Deep generative models, such as Generative Adversarial Networks (GANs), represent a more advanced solution. Instead of merely perturbing or mixing existing points, GANs learn the underlying probability distribution of the entire dataset, enabling them to generate novel, high-fidelity samples that better preserve the original data’s complex structure^41,47^.

While powerful, standard GANs can be difficult to train. They may suffer from issues like mode collapse and training instability. The WGAN-GP was developed specifically to address these challenges. By using the Wasserstein distance as a loss function and incorporating a gradient penalty, WGAN-GP promotes stable training and enhances the quality of the generated synthetic data, making it particularly suitable for complex tabular datasets^48,49^. It is important to distinguish these generative and regression-focused augmentation methods from other techniques. For instance, the well-known Synthetic Minority Over-sampling Technique also uses interpolation but was primarily designed to address class imbalance in classification problems, not for augmenting data in regression contexts.

## Methods

### Data acquisition and dataset composition

The dataset for this study was compiled from two distinct data collection phases. Ethical approval for all procedures was granted by the Ethics Committee of the Capital University of Physical Education and Sports (2022A57), and all participants provided written informed consent.

Initial data were sourced from a previously published study by Wang et al.^14^, involving 171 male participants with endurance rowing experience. In that foundational study, participants underwent a standardized 60-minute rowing ergometer test under one of eight randomized CPS conditions. These conditions featured CHO intakes from 0.50 to 1.20 g/kg/h, with a constant CHO-to-PRO ratio of 4:1. For each participant, 45 baseline indicators encompassing anthropometry, physiology, and lifestyle factors were recorded (Table 1), alongside total rowing distance. Full methodological details for this initial data acquisition are available in Wang et al.^14^. All methods were performed in accordance with the relevant guidelines and regulations.

**Table 1:** Summary of selected indicators across thirteen dimensions for personalized CPS recommendation model^14^.

Classification of indicators	Specific indicators
Living habits	Total cigarettes in last 30 days, Total alcohol units in last 30 days
Psychological status	Intuitive Eating Scale-2 (IES-2)
Sleep quality	Deep sleep, light sleep, rapid eye movement, Pittsburgh sleep quality index (PSQI)
Demographics	Age
Anthropometry	Height, weight, Triceps skinfold, subscapular skinfold, suprailiac skinfold, abdominal skinfold, upper arm circumference, waist circumference, hip circumference, subgluteal thigh circumference, mid-thigh circumference, calf circumference, Body water percentage, body fat percentage
Physical activity levels	Physical Activity Rating Scale-3 (PARS – 3)
Athletic ability	Left- and right-hand grip strength, average vertical jump height before exercise
Blood parameters	Blood glucose, blood lactate, hemoglobin (non-invasive test)
Central nervous system parameters	DC Potential
Cardiovascular system parameters	Resting heart rate, systolic blood pressure, diastolic blood pressure, Heart Rate Variability [HRV, (HF, LF, total power, SDNN, RMSSD, SDSD)]
Meal time	Previous meal time
Beverage ingredients	CHO, fat, sodium, magnesium, calcium
Sports performance	Rowing distance

Additional data were incorporated from a subsequent crossover validation study involving 12 male participants with endurance rowing experience, recruited using criteria identical to the initial phase. These participants each completed five trials of the same 60-minute rowing protocol under different nutritional strategies: PLA (no CHO or PRO), low-CHO (L-CHO; 0.80 g/kg/h CHO, 0 g/kg/h PRO), high-CHO (H-CHO; 1.00 g/kg/h CHO, 0 g/kg/h PRO), traditional CPS (T-CPS; 0.80 g/kg/h CHO, 0.20 g/kg/h PRO), and a personalized CPS (P-CPS). The P-CPS dosages (specific CHO and PRO g/kg/h) were determined for each of these 12 individuals using an initial ML model and enumeration method based on the aforementioned 171 datasets. For these 60 trials (12 participants × 5 conditions), the same 45 baseline indicators (Table 1) were obtained, along with rowing distance and the explicitly defined PRO intake rates for each condition.

### Data preprocessing

Following data acquisition, the data from both phases were consolidated and prepared for modeling. Specifically, the 171 records from the foundational study^14^ were combined with the 60 records generated from the subsequent crossover study (12 participants × 5 trial conditions), resulting in the final dataset of 231 records. Crucially, as both cohorts were recruited using identical criteria and drawn from the same population, the data from both acquisition phases are considered to be sampled from the same underlying population.

This dataset contained no missing values, providing a complete set of records for all subsequent analyses. Each record in this complete dataset was defined by 46 input features and one outcome variable (rowing distance). All 46 input features were numerical. These features encompassed direct physiological measurements (e.g., body weight), composite scores from validated scales (e.g., PSQI, PARS-3), and quantified behavioral data derived from questionnaires (e.g., total alcohol units consumed in the last 30 days). Although some behavioral inputs are derived from counts (e.g., number of days smoking), they represent quantities on a practical continuous scale and were thus appropriately treated as continuous variables in all subsequent analyses.

Feature scaling was not applied globally during preprocessing but was instead incorporated as an algorithm-specific step within the modeling pipeline, as detailed in Sect. 3.6.

### Overall study design and data partitioning

**Fig. 1:**
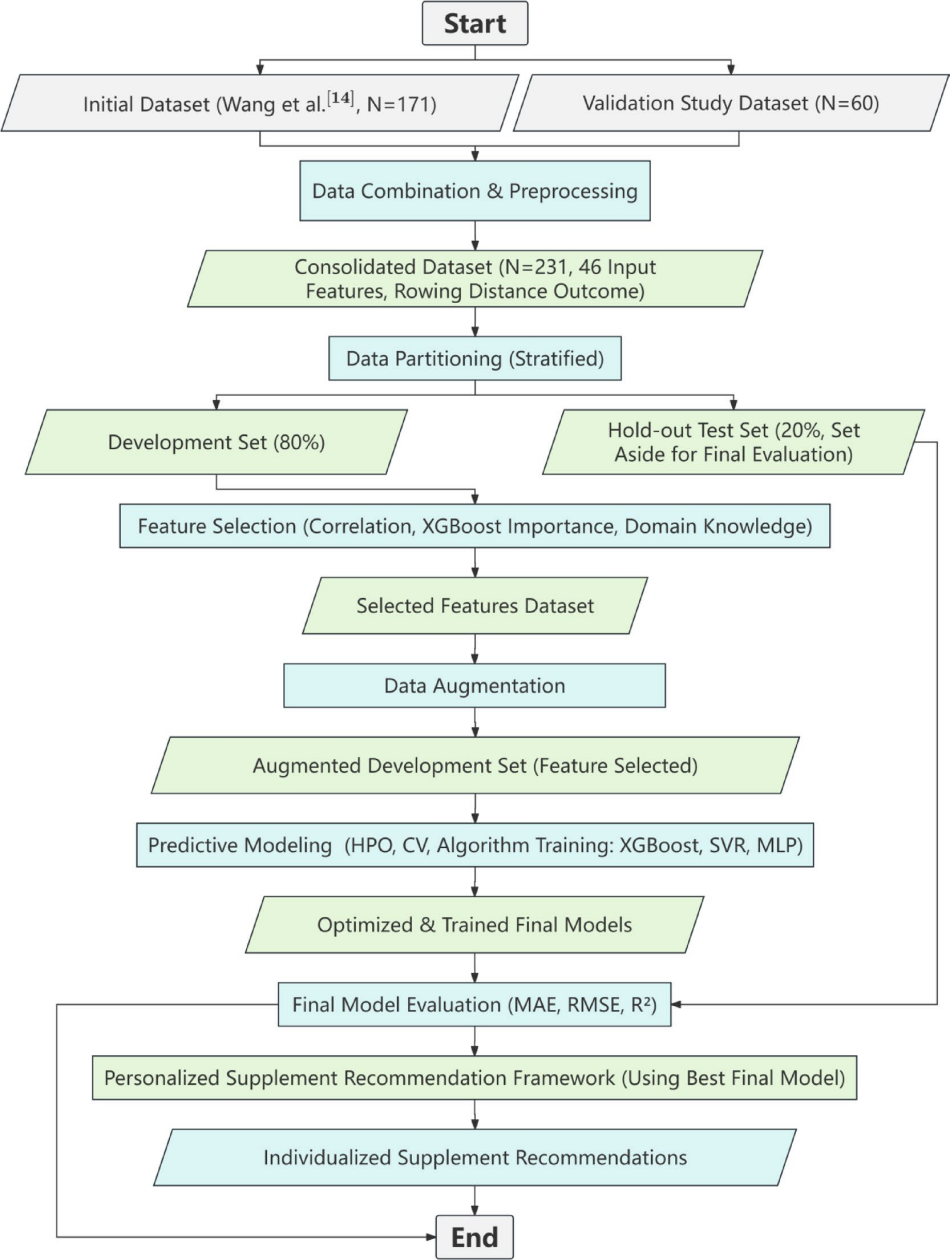
Overall study workflow diagram.

This study utilized a multi-stage ML framework, from initial data processing to final model validation (Fig. 1). The primary step involved stratifying this complete dataset into a development set (80%) and a final hold-out test set (20%). Stratification was performed based on the “Rowing distance” output variable, using four quartile-based bins. The final hold-out test set was rigorously isolated throughout all model development phases to ensure an unbiased evaluation of the model’s generalization capabilities. This strict separation is a fundamental strategy to test for overfitting, as it provides a final, unbiased assessment of model performance on entirely unseen data.

### Feature selection methodology

A primary objective of the modeling process was to mitigate the risk of overfitting, a notable concern given the dataset’s 231 trials relative to its 46 initial features. Therefore, to reduce model complexity and enhance generalization, this study employed a hybrid feature selection strategy. This approach integrates statistical analysis, model-based importance, and domain expertise to identify the most salient predictors. The multi-stage process began with correlation analysis to manage multicollinearity, a foundational step for model stability. Subsequently, an embedded method using an XGBoost model assessed the predictive contribution of each feature, a technique capable of capturing complex, non-linear relationships. Finally, domain knowledge was applied to ensure the selected features were not only statistically significant but also physiologically and nutritionally relevant. This hybrid methodology was chosen to balance computational efficiency, predictive power, and practical interpretability, offering a more comprehensive evaluation than using a single filter or a computationally expensive wrapper method alone (as discussed in Sect. 2.2).

#### Correlation analysis

Inter-feature relationships within the development set were quantified using the Pearson correlation coefficient (r). The Pearson coefficient measures the linear correlation between two features, x and y. It is calculated as:

1 $$r=\frac{{\sum \left( {{x_i} - \bar {x}} \right)\left( {{y_i} - \bar {y}} \right)}}{{\sqrt {\sum {{\left( {{x_i} - \bar {x}} \right)}^2}\sum {{\left( {{y_i} - \bar {y}} \right)}^2}} }}$$

A correlation matrix of all input features was computed. Feature pairs exhibiting an absolute Pearson correlation coefficient > 0.80 were identified as highly collinear. This analysis aimed to detect potential multicollinearity and feature redundancy.

#### Model-based feature importance assessment

An XGBoost regression model was employed to evaluate the predictive importance of each feature. Optimal hyperparameters for the XGBoost model were determined prior to importance assessment using a 5-fold CV procedure coupled with RandomizedSearchCV. Following HPO, the XGBoost model was trained on the entire development set. Feature importance scores were then extracted from this trained model.

#### Criteria for final feature subset selection

The final selection of the feature subset for subsequent modeling involved an integrated assessment. This assessment considered three sources of information: the correlation analysis results, the XGBoost-derived feature importance rankings, and established domain knowledge from exercise physiology and sports nutrition. Highly correlated features were reviewed; typically, one feature from a collinear pair was considered for removal, guided by its relative importance and theoretical relevance. Features with low importance scores were candidates for exclusion unless domain expertise strongly supported their retention.

### Data augmentation strategies

To address potential model limitations arising from a small sample size, this study evaluated several data augmentation techniques. A comparative approach was adopted, exploring methods that represent different levels of complexity and generative mechanisms. Random Noise Injection and Mixup were selected as computationally efficient baseline methods. They represent simple perturbation and interpolation strategies, respectively, and are commonly used for basic model regularization to improve model robustness and help prevent overfitting.

In contrast, WGAN-GP was chosen as an advanced generative model. Unlike the baseline methods, WGAN-GP is designed to learn the entire underlying distribution of the data, which can theoretically produce higher-fidelity synthetic samples that better preserve complex feature correlations. The WGAN-GP variant was specifically selected for its enhanced training stability and its ability to mitigate mode collapse, making it highly suitable for structured, non-image tabular data (as discussed in Sect. 2.3). While other interpolation methods like SMOTE exist, they are primarily designed to address class imbalance in classification tasks and were thus less appropriate for this regression context.

To ensure training stability of the WGAN-GP, all input data fed to the generator and critic, including the target variable, were temporarily standardized. After the synthetic data were generated, they were immediately transformed back to their original scale. Therefore, the augmented dataset provided to the downstream predictive models was on the same raw scale as the original data. The WGAN-GP architecture consisted of a generator and a critic. Both were multi-layer perceptrons using Adam optimizers (learning rate: 0.00005, *β*_*1*_ = 0.50, *β*_*2*_ = 0.90). The generator utilized ReLU activations and mapped a 100-dimension latent vector to the feature space. The critic used LeakyReLU activations. The model was trained to minimize the Wasserstein distance. A gradient penalty (coefficient *λ*_*gp*_ = 10.00) ensured critic training stability. The critic was updated five times per generator update. Training occurred for 10,000 epochs with a batch size of 32. Input data were standardized before training.

Mixup created synthetic samples by linearly interpolating pairs of randomly selected existing samples and their corresponding target values. For a pair of samples $\left( {{x_i},{y_i}} \right)$ and $\left( {{x_j},{y_j}} \right)$, a new sample $(\tilde {x},\tilde {y})$ was generated:

2 $$\tilde {x}=\lambda {x_i}+(1 - \lambda ){x_j}$$

3 $$\tilde {y}=\lambda {y_i}+(1 - \lambda ){y_j}$$

The interpolation coefficient λ was drawn from a Beta distribution, *Beta (α*,* α)*, with *α* = 0.20.

Random Noise Injection augmented data by adding Gaussian noise to the numerical features of randomly chosen existing samples. The noise added to each feature was sampled from a normal distribution with a mean of zero. The standard deviation of this noise was set to 5% of the original feature’s standard deviation.

For all augmentation methods, resulting numerical values were then clipped to a range slightly extended (1% of original range) from the original minimum and maximum of each feature, ensuring non-negativity for specific nutrients like CHO and PRO.

To enable a robust comparative evaluation, each of the three augmentation techniques was employed to generate a dataset of 2,000 synthetic samples from the original development set. Evaluation of data augmentation techniques involved comparing these newly generated datasets against the original data. This assessment included: Mann-Whitney U tests (MWU) of individual feature distributions (α = 0.05, Benjamini-Hochberg FDR correction); comparison of correlation matrices to assess inter-feature correlation structure preservation; and visual inspection of feature distribution similarity using Kernel Density Estimate (KDE) plots. The augmentation method demonstrating the most favorable overall preservation of these statistical and distributional characteristics was chosen for subsequent application.

### Predictive modeling pipeline

**Fig. 2:**
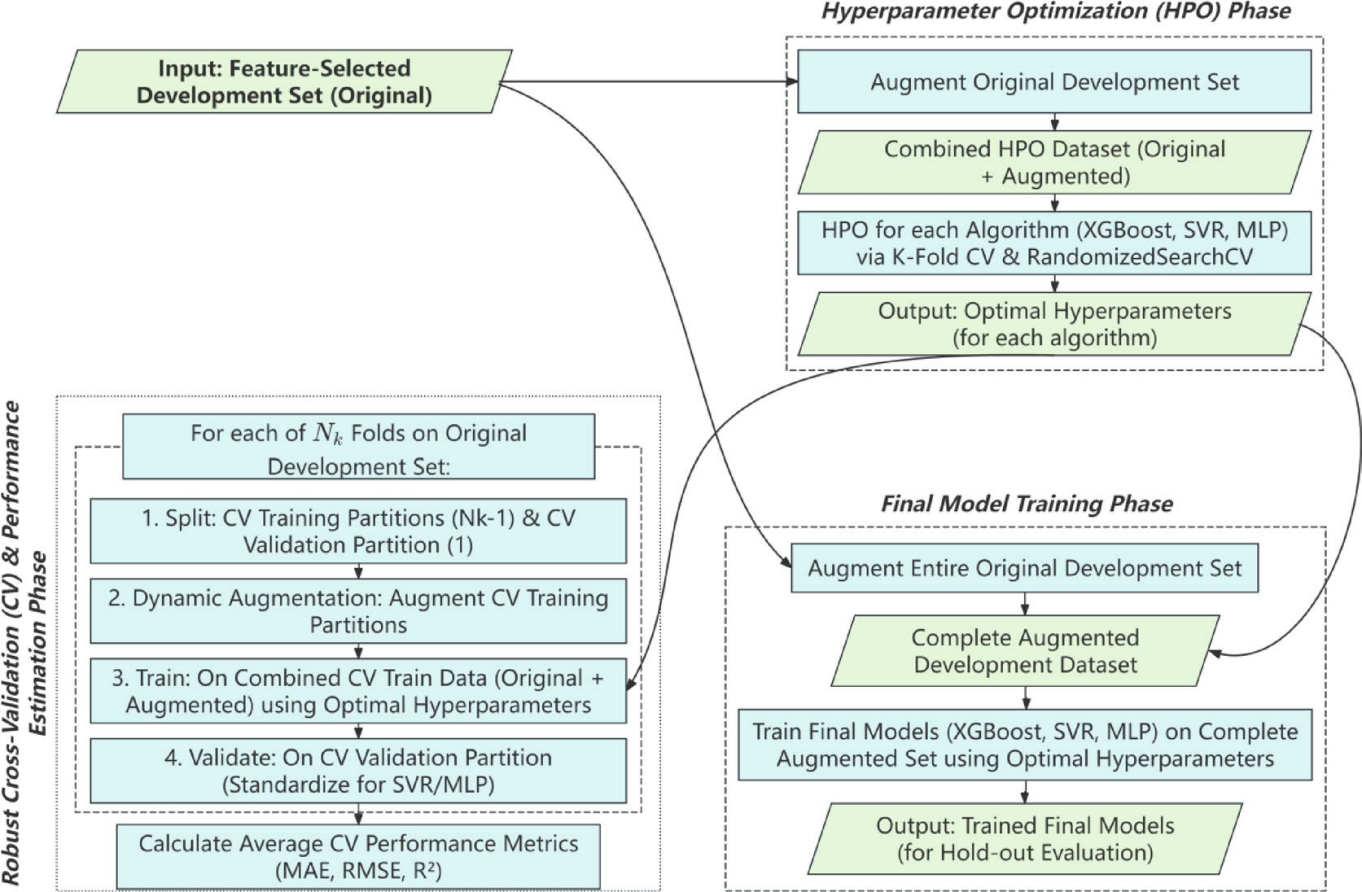
Detailed predictive model development and validation pipeline.

This section details the pipeline for developing and evaluating predictive models using the selected feature subset and the chosen optimal data augmentation strategy (Fig. 2).

#### Predictive algorithms

Three distinct regression algorithms were employed: XGBoost, Support Vector Regression (SVR), and a multi-layer perceptron (MLP) neural network. XGBoost was selected for its high efficiency and predictive accuracy with tabular data^50,51^. SVR was chosen for its effectiveness in high-dimensional spaces and its flexibility with different kernel functions^52,53^. The MLP was included for its ability to model complex non-linear relationships^54,55^.

As a tree-based ensemble method, XGBoost is insensitive to the scale of input features. Therefore, no feature scaling was applied when developing the XGBoost models. For the SVR models, a StandardScaler was integrated into the modeling pipeline using a scikit-learn Pipeline object. This step standardized the input features (X) only, while the target variable (Rowing distance) remained on its original scale. This process was applied consistently for models trained with and without augmented data. For the MLP models, both the input features (X) and the target variable (y) were independently standardized. Two separate StandardScaler instances were fitted on the training data for features and the target, respectively, before they were fed into the network. For predictions, the model’s output was transformed back to the original scale using the fitted scaler for the target variable.

#### Common model development framework (on development set)

A unified framework was established for the development and validation of all three regression models (XGBoost, SVR, MLP). This process, conducted entirely on the development set, involved two key stages to identify optimal hyperparameters and assess model stability.

- Stage 1: Augmentation for Hyperparameter Optimization (HPO). To create a robust dataset for HPO, the original development set was augmented by generating a synthetic dataset equal in size (a 1:1 augmentation ratio). HPO for each algorithm was then conducted on this combined dataset (original + synthetic), which effectively doubled the number of samples available for the tuning process.
- Stage 2: Dynamic Augmentation for Cross-Validation (CV). To evaluate model performance with the optimized hyperparameters, a 5-fold CV was performed on the original development set to ensure a robust evaluation of model generalization and minimize the risk of overfitting to any single data partition. Critically, within each fold, the training partition was dynamically augmented by generating synthetic samples equal in size to that partition (a 1:1 ratio). Each model was then trained on the combined data (original CV training partition + its synthetic counterpart) and validated on the untouched, original CV validation partition.

This multi-stage augmentation strategy systematically expanded the dataset to enhance model training and validation, with the specific sample sizes summarized in Table 2.

**Table 2:** Summary of the data augmentation strategy and sample sizes at different modeling stages.

Modeling stage	Input data (Original samples)	Generated synthetic samples	Augmentation ratio (Synthetic: Original)	Total training samples
HPO	184 (Full Development set)	184	1:01	368
5-Fold CV (per fold)	~ 147 (4/5 of development set)	~ 147	1:01	~ 294
Final model training	184 (Full development set)	368	2:01	552

The final hold-out test set, consisting of 47 samples, was kept separate and was not used in any augmentation or training stages.

Performance within this CV framework was assessed using Mean Absolute Error (MAE), Root Mean Squared Error (RMSE), and the coefficient of determination (*R*^*2*^).

#### Hyperparameter optimization

To identify the optimal hyperparameters for each predictive algorithm (XGBoost, SVR, and MLP), a systematic hyperparameter optimization (HPO) process was conducted. This process was performed on the development set, which was augmented at a 1:1 ratio as described in Sect. 3.6.2.

For the XGBoost and SVR models, a Randomized Search Cross-Validation (RandomizedSearchCV) strategy was employed. This process was configured with 50 search iterations (n_iter = 50) and a 5-fold cross-validation (cv = 5) scheme. For the MLP model, a similar random search was manually implemented over 25 iterations, also utilizing a 5-fold CV framework for robust evaluation. Across all models, the negative mean absolute error (neg_mean_absolute_error) was used as the scoring metric to guide the search towards the best-performing parameter set. The randomized search approach was chosen for its computational efficiency, as it allows for a broad exploration of the parameter space without the exhaustive cost of a grid search. The specific hyperparameters and their corresponding search spaces for each model are detailed in Table 3.

**Table 3:** Hyperparameter search spaces for model optimization.

Model	Hyperparameter	Search space / Values
XGBoost	n_estimators	[100, 200, 300, 400, 500]
	max_depth	[3, 5, 7, 9]
	learning_rate	[0.01, 0.05, 0.1, 0.15, 0.2]
	subsample	[0.7, 0.8, 0.9, 1.0]
	colsample_bytree	[0.7, 0.8, 0.9, 1.0]
	gamma	[0, 0.1, 0.2, 0.3]
	reg_alpha	[0, 0.01, 0.1, 0.5, 1.0]
	reg_lambda	[0.5, 1.0, 1.5, 2.0]
SVR	kernel	[‘rbf’, ‘linear’, ‘poly’]
	C	[0.1, 1, 10, 50, 100, 200, 500]
	gamma	[‘scale’, ‘auto’, 0.001, 0.005, 0.01, 0.05, 0.1]
	epsilon	[0.01, 0.05, 0.1, 0.15, 0.2, 0.3, 0.5]
	degree	[2, 3, 4]
MLP	hidden_dims	[[32], [64], [32, 16], [64, 32], [128, 64], [128, 64, 32]]
	learning_rate	[0.0005, 0.001, 0.005]
	batch_size	[16, 32, 64]
	dropout_rate	[0.2, 0.3, 0.4, 0.5]
	weight_decay	[1e-5, 1e-4, 5e-4, 1e-3, 2e-3]

### Final model training and evaluation

Following the development and validation phase, a definitive model for each algorithm was trained and subsequently evaluated on the independent hold-out test set. To facilitate a fair comparison, this process was conducted in parallel for models trained with and without data augmentation.

#### Final model training

Two sets of final models were trained using the optimized hyperparameters identified during HPO for each algorithm (XGBoost, SVR, and MLP).

- Final Baseline Models (without augmentation): For each algorithm, a final baseline model was trained on the entire original development set (184 samples).
- Final Augmented Models: Concurrently, a second set of models was trained. The entire original development set was first augmented by generating a synthetic dataset equivalent to twice its size (a 2:1 augmentation ratio). The final augmented models were then trained on the comprehensive combined dataset (552 samples).

For the SVR and MLP models, the algorithm-specific scaling protocols, as described in Sect. 3.6.1, were applied to this comprehensive training dataset.

#### Evaluation on the final hold-out test set

The generalization ability of all trained final models (both baseline and augmented) was assessed on the previously segregated final hold-out test set. This test set was not used in any preceding training, augmentation, or HPO stages. Performance on this hold-out set was quantified using MAE, RMSE, and R², providing the definitive, unbiased measure of each model’s predictive capability and allowing for a direct comparison of the impact of data augmentation.

### Personalized supplement recommendation framework

Individualized supplement recommendations were generated using the trained predictive model. This process determined the optimal supplementation strategy (either CPS or CHO-only) and intake rates for each participant. The participant’s unique baseline indicators, as used in the predictive model, remained constant during this procedure.

**Fig. 3:**
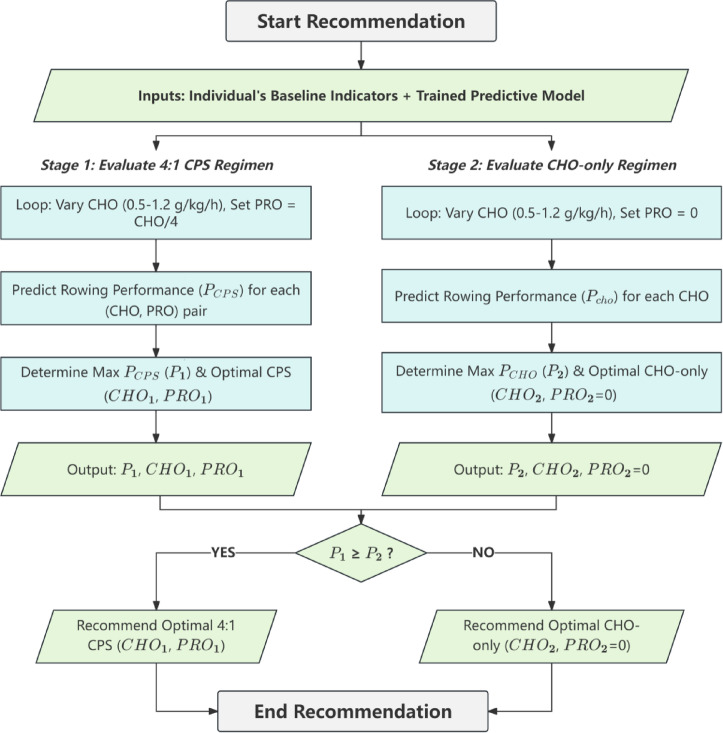
Personalized supplement recommendation framework flowchart.

The framework involved a two-stage evaluation (Fig. 3). In the first stage, optimal intake rates for a 4:1 CPS were determined. CHO intake was systematically varied from 0.50 to 1.20 g/kg/h. This range used 0.01 g/kg/h increments, creating 71 distinct levels. For each CHO level, PRO intake was set at a 4:1 ratio (PRO = CHO/4). Each CHO and PRO combination, along with the participant’s constant baseline indicators, was input into the predictive model. This yielded 71 performance predictions. The CHO and PRO intake rates corresponding to the maximum predicted performance (P_1_) defined the optimal 4:1 CPS regimen for that individual.

In the second stage, optimal intake rates for a CHO-only supplement were identified. CHO intake was again varied across the same 71 levels (0.50 to 1.20 g/kg/h). For these evaluations, PRO intake was consistently set to 0 g/kg/h. The predictive model then generated another 71 performance predictions based on these CHO-only inputs and the participant’s baseline indicators. The CHO intake rate (with PRO = 0 g/kg/h) associated with the maximum predicted performance (P_2_) defined the optimal CHO-only regimen.

Finally, the personalized supplement recommendation was determined by comparing P_1_ and P_2_. If P_1_ was greater than or equal to P_2_, the optimal 4:1 CPS regimen was recommended. If P_2_ was greater than P_1_, the optimal CHO-only regimen was recommended. This approach ensured selection of the strategy predicted to yield the highest performance for each individual.

## Results

### Feature selection

**Fig. 4:**
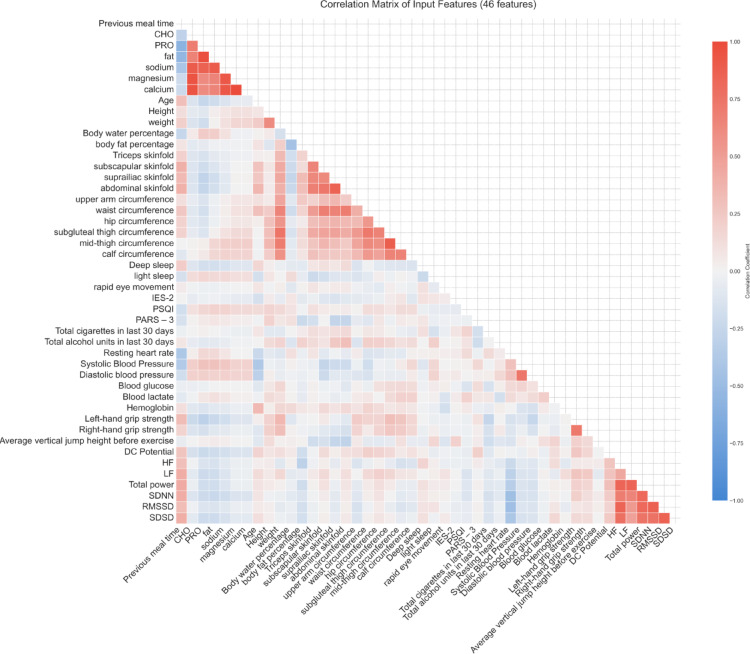
Correlation matrix of input features.

**Fig. 5:**
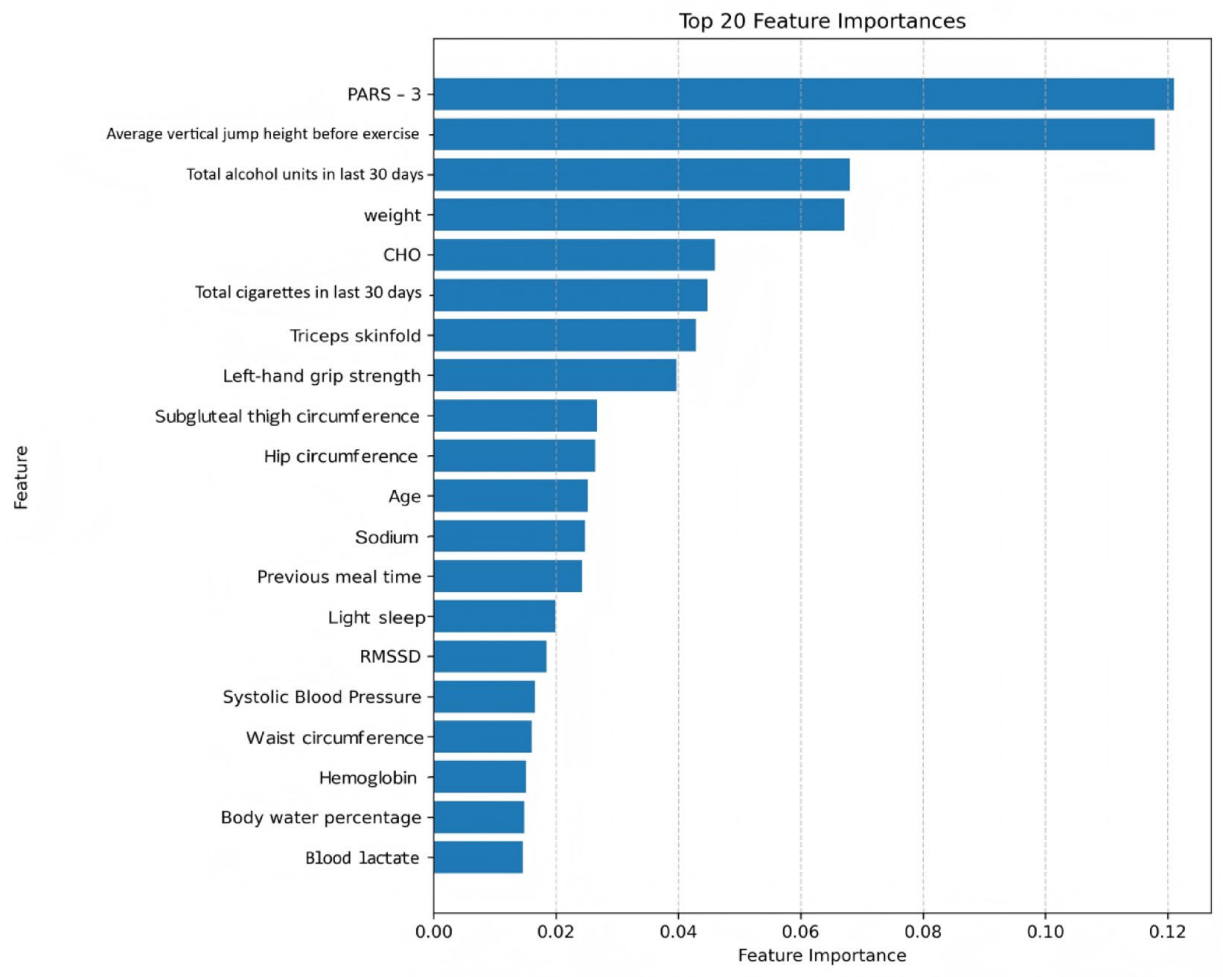
The top 20 feature importances of XGBoost.

Feature selection refined the initial 46 input features. The correlation analysis identified 18 feature pairs with an absolute Pearson correlation coefficient greater than 0.80, indicating high collinearity (Table 4; Fig. 4). Feature importance rankings, derived from an XGBoost model, are presented in Fig. 5.

**Table 4:** Highly correlated feature pairs.

Feature 1	Feature 2	Correlation coefficient
Magnesium	Calcium	1.00
PRO	Fat	0.97
CHO	Magnesium	0.95
CHO	Calcium	0.95
Sodium	Magnesium	0.92
Sodium	Calcium	0.92
fat	Sodium	0.90
CHO	Sodium	0.90
Subgluteal thigh circumference	Mid-thigh circumference	0.88
SDNN	RMSSD	0.86
PRO	Sodium	0.86
RMSSD	SDSD	0.86
suprailiac skinfold	Abdominal skinfold	0.86
HF	RMSSD	0.85
LF	Total power	0.85
HF	Total power	0.84
Total power	SDNN	0.82
Total power	RMSSD	0.81

An integrated assessment of these correlations, feature importances, and domain knowledge resulted in the selection of a final subset of 21 features. The selected features were: PARS – 3, average vertical jump height before exercise, Total alcohol units in last 30 days, weight, CHO, Total cigarettes in last 30 days, Triceps skinfold, Left-hand grip strength, subgluteal thigh circumference, hip circumference, Age, Previous meal time, light sleep, RMSSD, systolic blood pressure, waist circumference, hemoglobin, Body water percentage, blood lactate, PRO, and Blood glucose. The XGBoost model’s optimized hyperparameters and performance, pre- and post-feature selection, are presented in Table 5.

**Table 5:** XGBoost model hyperparameters and performance before and after feature selection.

Category	Parameter/Metric	Before feature selection	After feature selection
Hyperparameter tuning	Optimal CV score		
	(neg_MAE)	-627.79	-555.41
Optimal hyperparameters	Subsample	1	0.9
	reg_lambda	0.5	0.5
	reg_alpha	0.5	1
	n_estimators	200	500
	max_depth	7	5
	Learning_rate	0.1	0.05
	Gamma	0.1	0.2
	colsample_bytree	0.8	0.9
Average train performance	MAE	104.22	68.66
	RMSE	122.10	90.90
	R^2^ score	0.96	0.98
Average test performance	MAE	643.80	587.65
	RMSE	810.90	742.98
	R^2^ score	0.32	0.42

### Evaluation of data augmentation methods

**Fig. 6:**
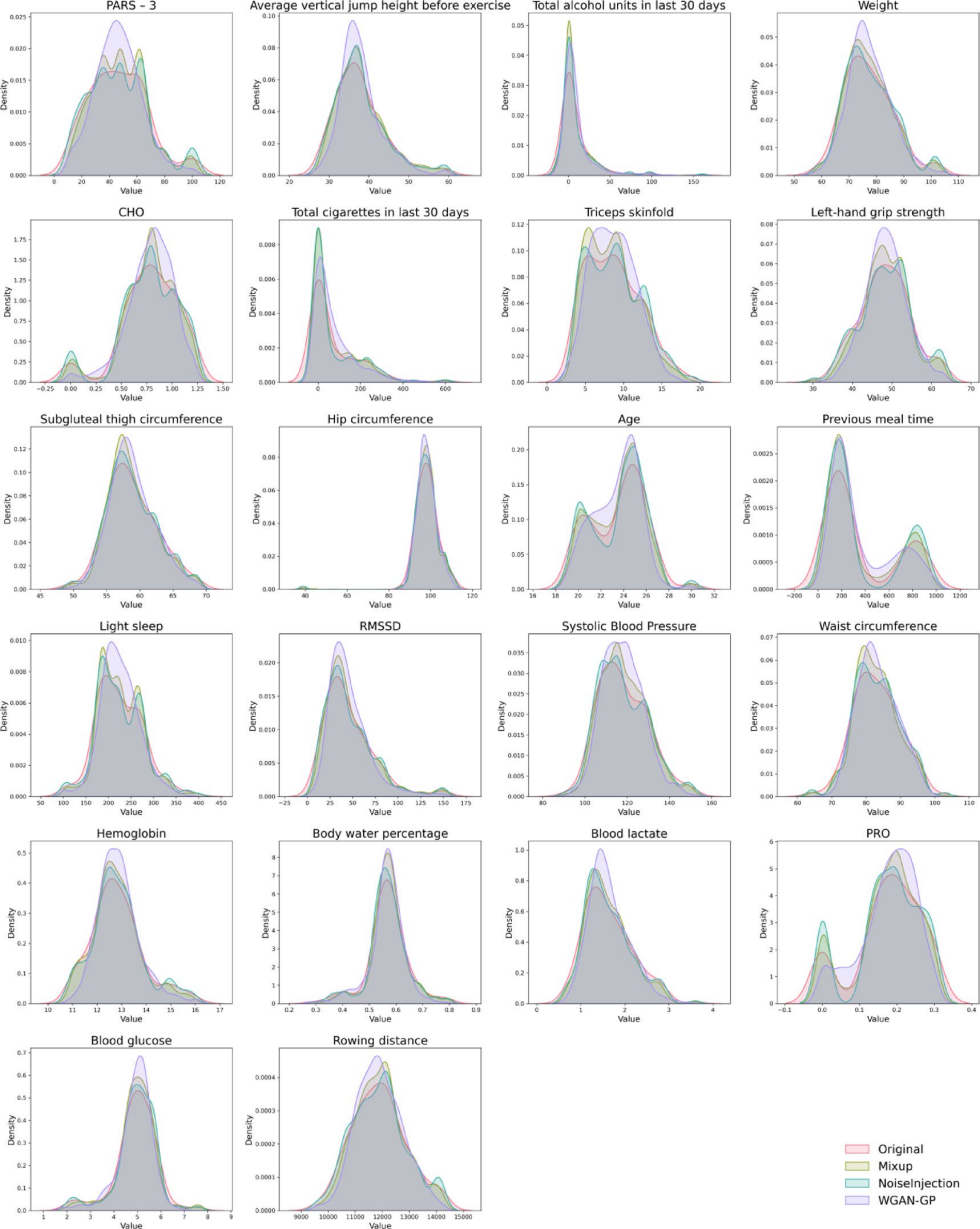
KDE plots of selected feature distributions. Original (red line), Mixup (green line), NoiseInjection (blue line), and WGAN-GP (purple line). Each subplot represents one feature, with values on the x-axis and probability density on the y-axis.

As can be seen in the Fig. 6, WGAN-GP distributions demonstrated the closest visual match to Original distributions across the majority of features. This alignment encompassed distributional shape, modality, and spread. For features with complex patterns, such as bimodal or highly skewed distributions, WGAN-GP also showed high concordance with Original distributions. Mixup distributions appeared consistently flatter and wider than Original distributions. NoiseInjection distributions exhibited variable similarity to Original distributions, with some deviations in shape or modal alignment.

**Fig. 7:**
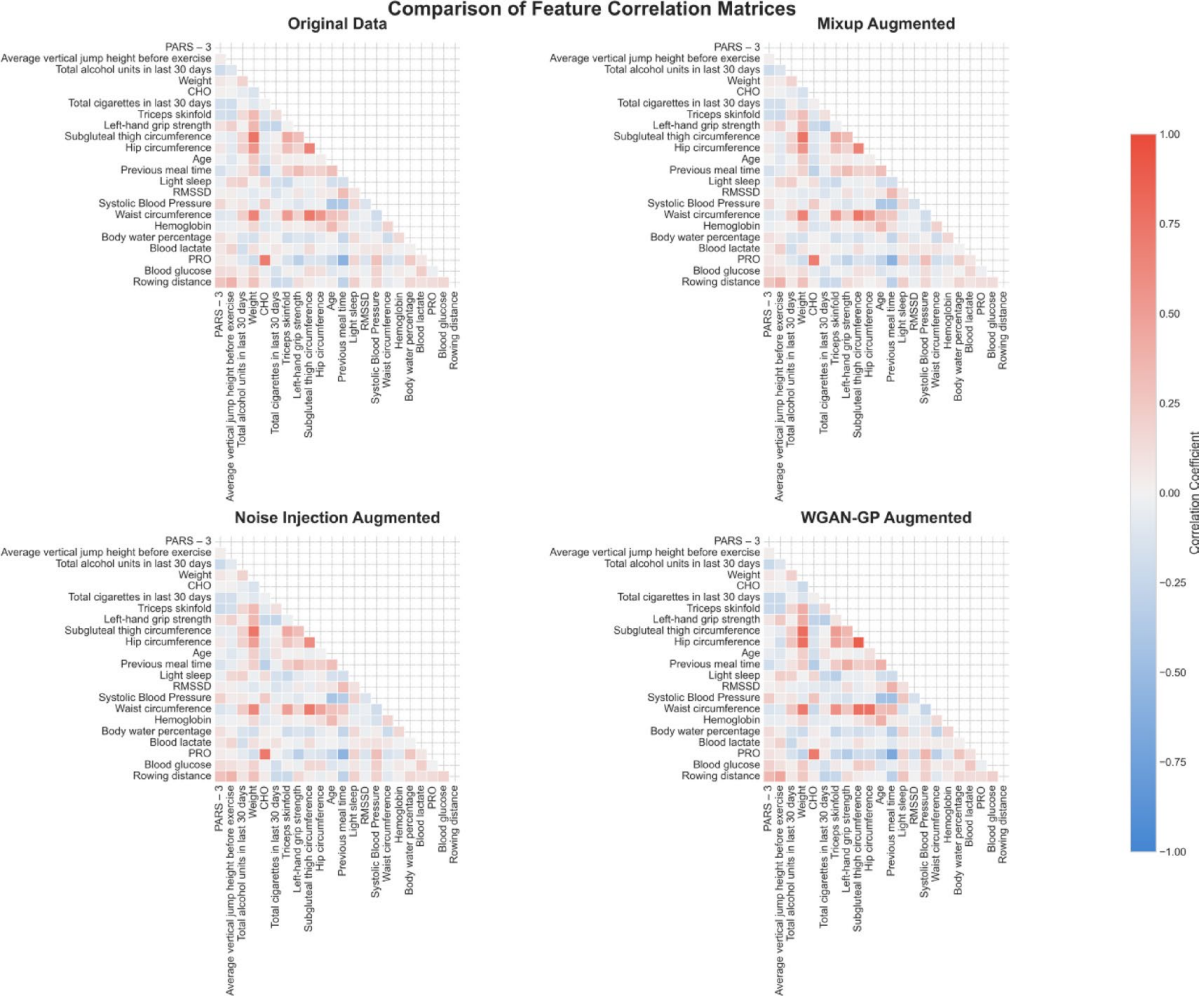
Comparison of inter-feature correlation matrices. Red cells indicate positive correlations, blue cells indicate negative correlations, and the color intensity corresponds to the magnitude of the correlation coefficient.

The preservation of inter-feature correlation structures by different augmentation methods was visually evaluated (Fig. 7). The correlation matrix from the Mixup augmented dataset displayed a general attenuation of correlation magnitudes relative to the Original dataset; many correlations appeared noticeably weaker. In contrast, both the Noise Injection and WGAN-GP augmented datasets substantially preserved the overall patterns and strengths of the inter-feature correlations found in the Original dataset. The WGAN-GP augmented dataset, in particular, closely mirrored the nuanced correlation structure of the Original data.

**Fig. 8:**
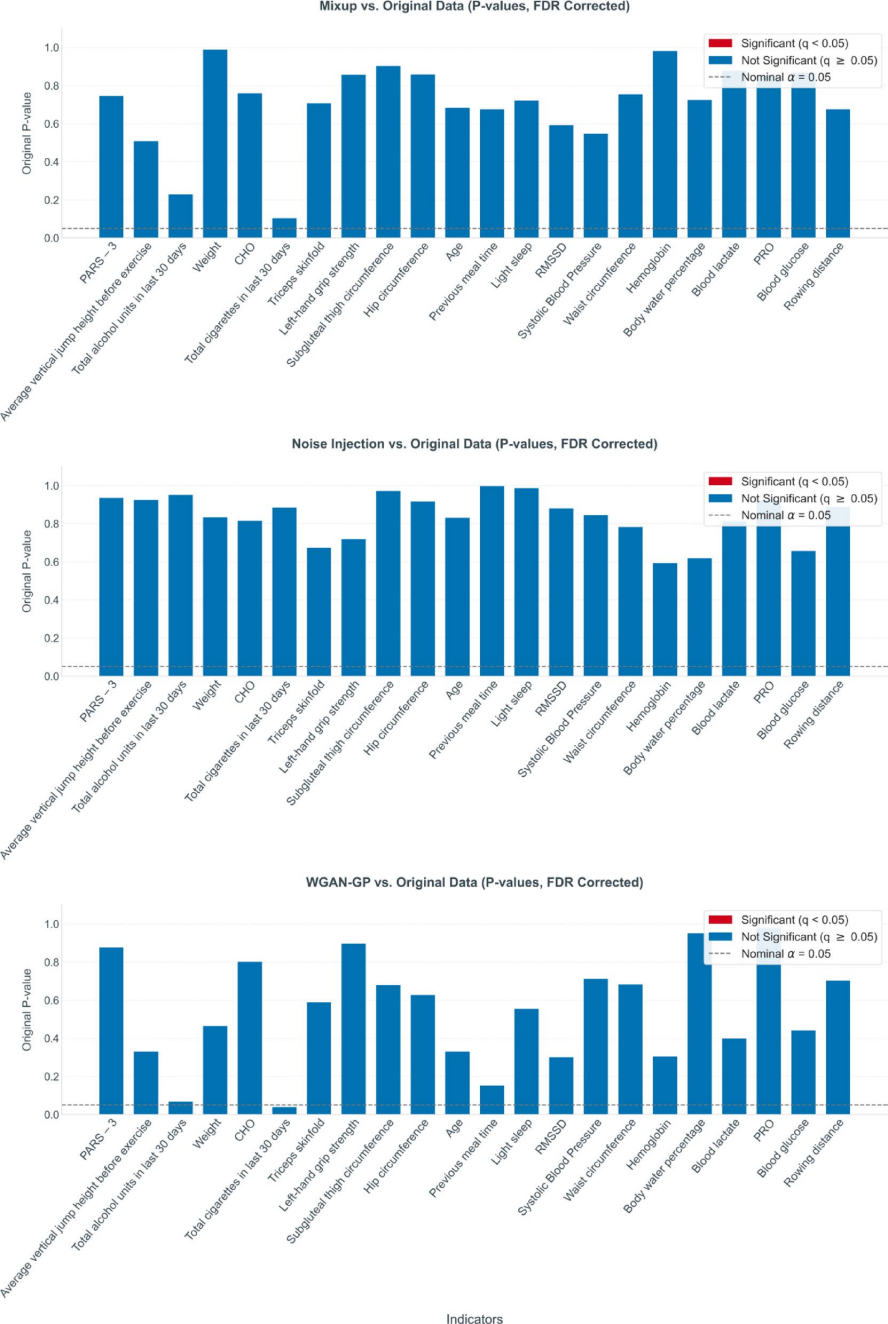
Statistical comparison of indicator distributions between augmented and original datasets via MWU. Each subplot compares an augmented dataset (Mixup, noise injection, or WGAN-GP) against the original dataset. Bars represent the original (uncorrected) p-values from MWU for each indicator. Bar colors indicate statistical significance after FDR correction: red for significant (q < 0.05), blue for not significant (q ≥ 0.05). The horizontal dashed line denotes the nominal alpha level of 0.05.

Further quantitative comparisons using MWU tests assessed feature distributions from augmented (Mixup, NoiseInjection, WGAN-GP) against Original datasets. Following Benjamini-Hochberg FDR correction, all q-values for the 21 input features and the output variable exceeded 0.05 (Fig. 8), indicating no statistically significant differences.

Consequently, WGAN-GP was chosen as the data augmentation technique for this study due to its superior overall performance in preserving data characteristics.

### Predictive modeling performance

The comprehensive performance evaluation of all predictive models, both with and without data augmentation, is presented in Table 6. On the original dataset, the MLP model yielded the highest predictive accuracy on the final hold-out test set (R^2^ = 0.57). However, its performance during development was less consistent, as indicated by a lower CV validation score (R^2^ = 0.33). In contrast, the baseline XGBoost model, while achieving a slightly lower test performance (R^2^ = 0.48), demonstrated superior stability during CV (R^2^ = 0.42).

Data augmentation with WGAN-GP substantially improved the XGBoost model’s generalization ability. The augmented XGBoost model became the top performer on the hold-out test set (R^2^ = 0.53, RMSE = 715.97 m). This model also maintained stable performance metrics during the development phase. The SVR models consistently registered the lowest predictive accuracy across all tested conditions. Consequently, the XGBoost model trained with augmented data was identified as the most effective and robust predictor of rowing performance.

**Fig. 9:**
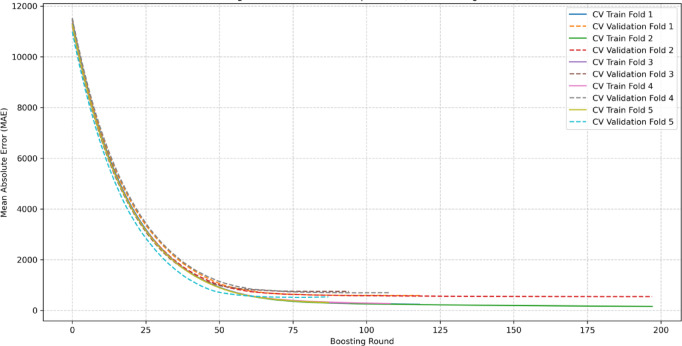
MAE learning curves for the final XGBoost model during 5-fold CV on the WGAN-GP augmented development set.

**Fig. 10:**
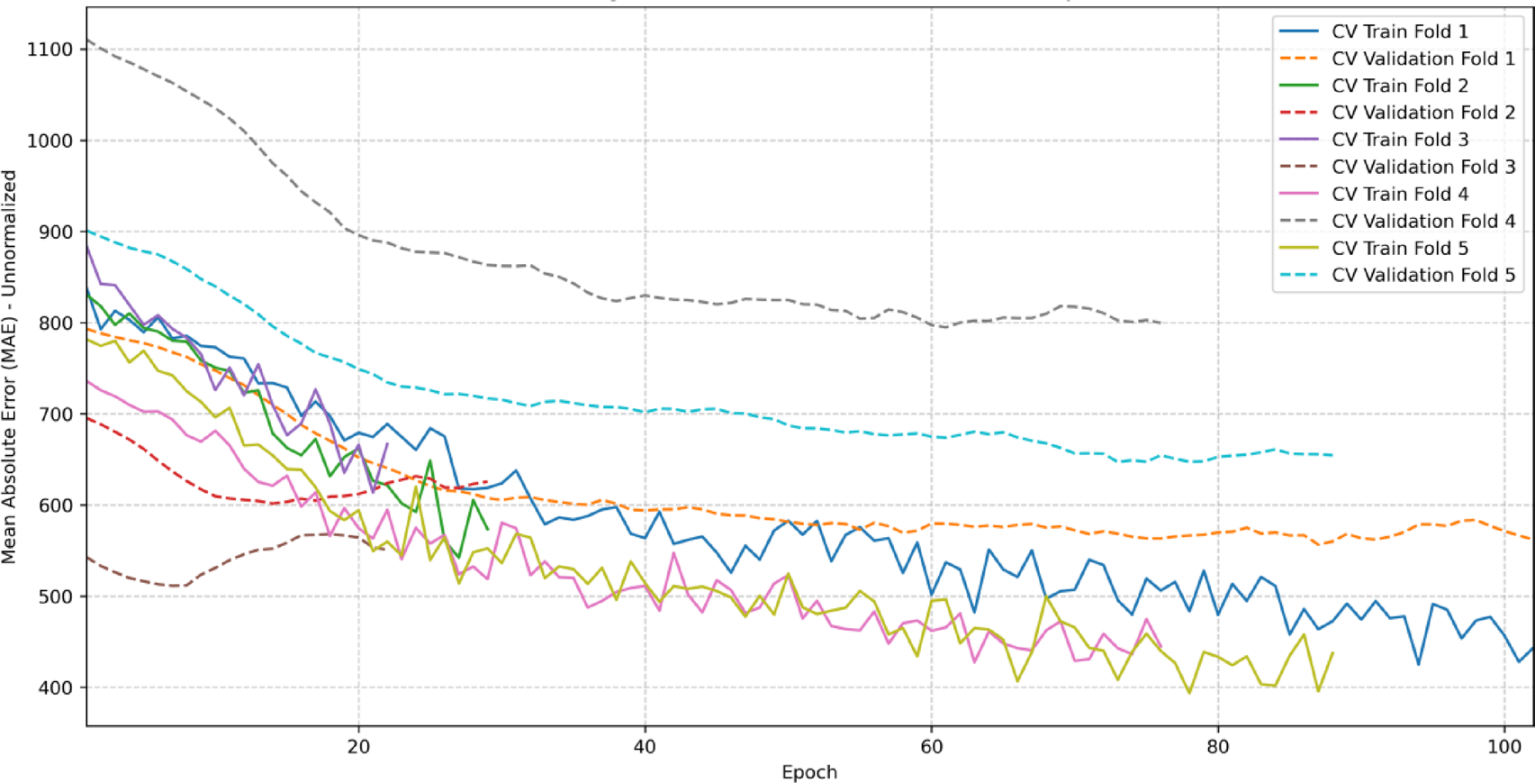
Training and validation MAE per fold for the baseline MLP model.

The stability of this final augmented XGBoost model is visually confirmed by its MAE learning curves, which show consistent convergence across the 5 validation folds (Fig. 9). Conversely, the baseline MLP model displayed significant performance variance across the same CV process, suggesting training instability (Fig. 10).

**Table 6:** Comparison of model performance on the test set with and without data augmentation.

Model	Condition	Key optimized hyperparameters	CV performance (on development set)					Final performance (on hold-out test set)		
			CV train MAE	CV train R^2^	CV valid MAE	CV valid RMSE	CV valid R^2^	Test MAE	Test RMSE	Test R^2^
XGBoost	Baseline	n_estimators = 500, max_depth = 5, learning_rate = 0.05	68.66	0.98	587.65	742.96	0.42	642.40	751.26	0.48
	**Augmented**	n_estimators = 400, max_depth = 3, learning_rate = 0.05	276.98	0.84	614.90	759.11	0.39	**632.05**	**715.97**	**0.53**
SVR	Baseline	kernel=’poly’, C = 500, degree = 3	356.48	0.65	644.75	830.19	0.24	620.12	773.31	0.45
	Augmented	kernel=’rbf’, C = 500, gamma=’auto’	284.18	0.73	660.34	840.97	0.26	644.96	765.48	0.46
MLP	**Baseline**	hidden_dims=[32, 16], learning_rate = 0.001, batch_size = 16	434.94	0.71	638.54	785.69	0.33	**556.96**	**686.76**	**0.57**
	Augmented	hidden_dims=[128, 64], learning_rate = 0.005, batch_size = 16	256.23	0.86	706.37	868.69	0.20	641.48	771.49	0.46

Note: Bold values highlight the metrics of the best-performing model on the hold-out test set for each data condition (Baseline and Augmented).

**Fig. 11:**
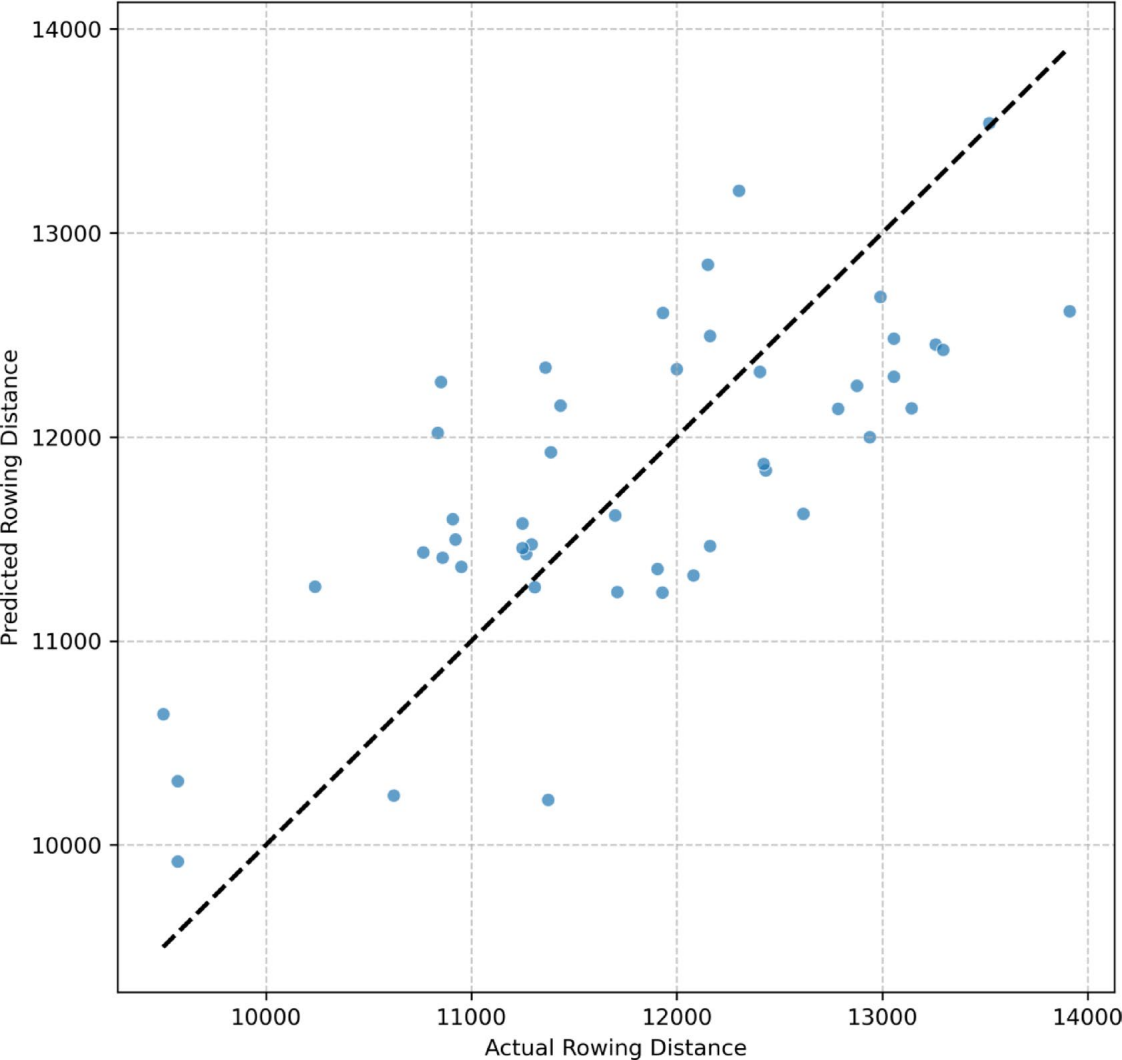
Correlation between actual and model-predicted rowing distance on the hold-out test set for the final XGBoost model.

The final predictive accuracy of the augmented XGBoost model on the hold-out test set is depicted in Fig. 11. The scatter plot shows a clear positive linear relationship between the model-predicted and actual rowing distances, with data points clustered around the line of identity.

Comprehensive performance visualizations for all model configurations are available in the Supplementary Materials. These materials contain the complete set of CV learning curves for each model, trained on both the original and augmented datasets. Scatter plots detailing the predictive performance of each model variant on the final hold-out test set are also provided.

**Fig. 12:**
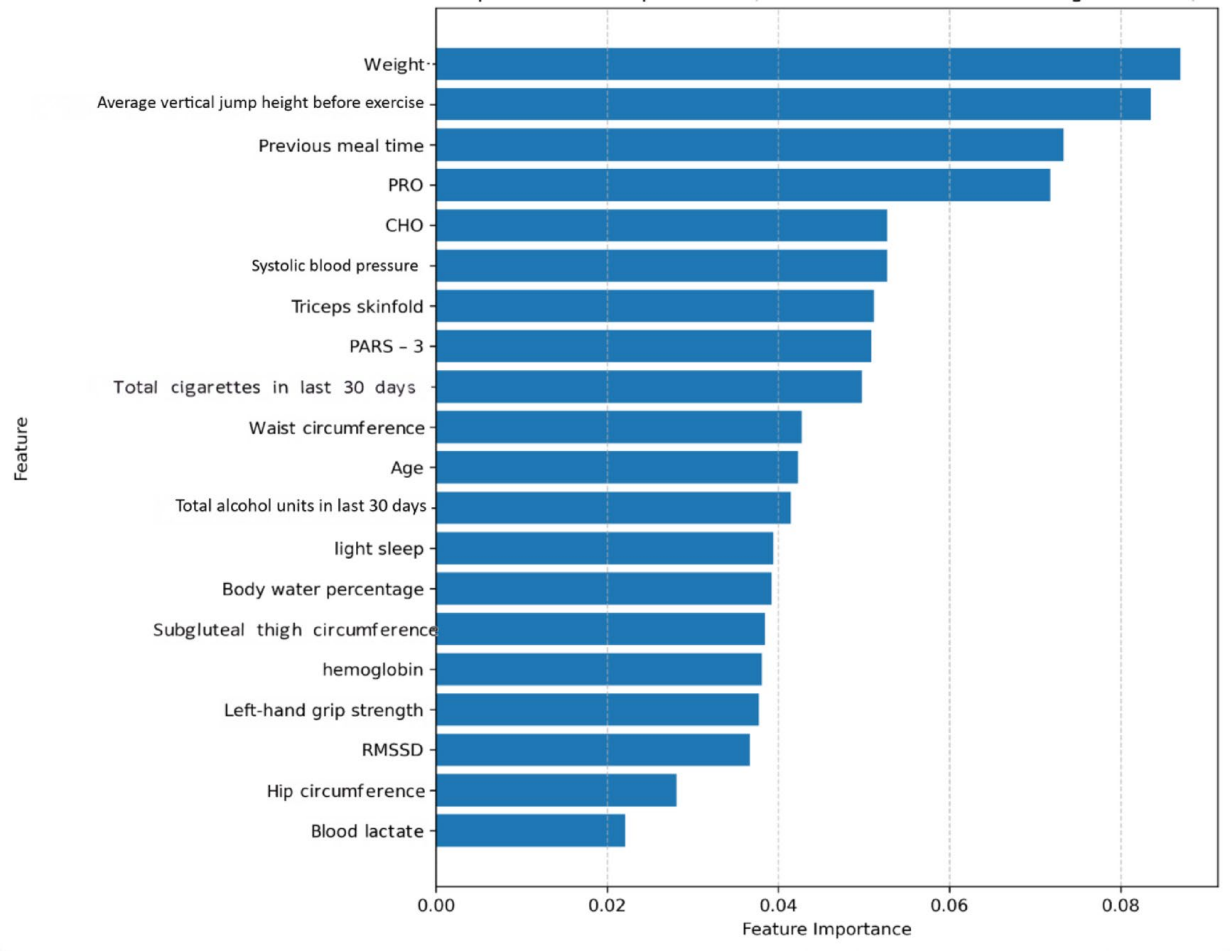
Top 20 feature importances from the final XGBoost model (Trained with WGAN-GP augmentation).

Figure 12 illustrates the 20 most influential features according to this model. Weight was the most important feature, followed closely by average vertical jump height before exercise. Previous meal time, PRO, and CHO also ranked within the top five most important features. Among the top 20 features displayed, blood lactate and hip circumference registered the lowest importance scores.

## Discussion

This study demonstrates the effective application of an integrated ML strategy for predicting endurance rowing performance. A multi-faceted feature selection process successfully distilled critical performance indicators from a broad initial set. Notably, data augmentation using WGAN-GP substantially enhanced the predictive accuracy of an optimized XGBoost model. These findings underscore the utility of advanced computational methods for addressing data limitations common in sports science. Furthermore, this approach establishes a robust foundation for developing data-driven, personalized athletic support strategies.

This research builds upon foundational exploratory work that established the initial feasibility of predicting rowing performance with a smaller datase^56^. The present study transitions from exploration to methodological validation by introducing several significant advancements. First, it implements a systematic, hybrid feature selection strategy, ensuring that subsequent modeling is founded on the most salient physiological and nutritional predictors. More critically, this study provides a comprehensive and comparative evaluation of data augmentation techniques, directly addressing the sample size limitations inherent in the prior work. By validating an WGAN-GP against simpler baseline methods, this research offers a validated solution to a persistent challenge in the field. Finally, all models were developed using a robust CV protocol and assessed with multiple metrics (R², RMSE, MAE), providing a more thorough and reliable assessment of generalization than the initial exploratory study.

This study highlights the efficacy of a hybrid feature selection strategy in refining complex sports science datasets. Such strategic dimensionality reduction streamlined the initial feature set. It also enhanced the predictive performance of the subsequent XGBoost model, a benefit consistent with research showing improved model outcomes with appropriately selected features^57,58^. The feature selection process also effectively managed multicollinearity by excluding redundant variables. This ensured a more parsimonious final model, improving interpretability and efficiency, a recognized benefit of careful feature selection^58^.

The selection of WGAN-GP as the data augmentation strategy underscores its capacity for high-fidelity tabular data synthesis in sports science. WGAN-GP models can effectively capture complex data distributions and inter-feature dependencies^59,60^. This capability likely explains its superior preservation of the original dataset’s characteristics, which was evident in the visual assessments via KDE plots and correlation matrices. While MWU indicated no statistically significant distributional shifts for any individual feature across the augmentation methods (all q > 0.05), these univariate statistics alone may not fully reflect overall data realism. The importance of comprehensive validation for synthetic data, extending beyond simple statistical tests, is well-established^61^.

Therefore, WGAN-GP was chosen due to its superior performance in these more holistic qualitative assessments. Simpler methods like basic Mixup, which rely on linear interpolations^45^, might not adequately model the non-linear relationships often present in physiological data. This could lead to less representative synthetic samples. Similarly, while Noise Injection has shown utility in some contexts^43,44^, its effectiveness is highly domain-dependent; inappropriately applied noise can even obscure important data patterns or mimic irrelevant phenomena^62^. The documented success of WGAN-GP in generating quality synthetic data that enhances predictive modeling in other complex domains further supported its selection for this study^60,63^.

The impact of WGAN-GP data augmentation on model generalization was algorithm-dependent. It yielded notable performance gains for the XGBoost and SVR models, suggesting that the synthetic data provided a richer feature space for these algorithms to learn from^64,65^. Conversely, the MLP model’s performance decreased after augmentation. This suggests that the baseline MLP may have overfitted to specific patterns in the small original dataset, and the augmented data, by introducing more variability, acted as a regularizer that prevented this overfitting but reduced its score on the specific hold-out test set. This highlights a critical finding: data augmentation does not universally guarantee improved performance and its effectiveness must be evaluated on a model-by-model basis.

The XGBoost algorithm, when combined with WGAN-GP augmentation, emerged as the most robust and well-rounded model in this study. XGBoost’s strong predictive capabilities and robustness are well-documented across various complex predictive tasks^64,66,67^. Its ensemble nature, which iteratively refines predictions by learning from the errors of preceding models^68^, may effectively harness the richer and more diverse data space provided by WGAN-GP. The successful application of GAN-enhanced XGBoost models in other domains further supports this synergy^69^. The final R² value of 0.53 achieved by this combination indicates that the model accounts for a substantial portion of the variance in rowing distance. While not capturing all variability inherent in complex human athletic performance, this level of predictive accuracy offers practical value. It can guide personalized interventions and enhance the understanding of key performance determinants.

Despite the benefits of WGAN-GP augmentation on final test set generalization, a noticeable gap between CV training and validation performance persisted across all models. This suggests that some overfitting tendencies remained during the model development phase. Such challenges can arise when modeling complex physiological systems where true sample diversity may not be fully captured even by synthetic data expansion^70^, or where imbalanced representation of certain data characteristics exists^71^. While data augmentation addresses issues of data scarcity and can reduce overfitting^70,72^, the inherent complexity of predicting athletic performance may necessitate additional strategies. Future research could explore advanced regularization techniques^73,74^. Additionally, model architectures promoting sparsity and robustness may further mitigate overfitting and enhance generalizability.

The feature importance analysis from the final WGAN-GP augmented XGBoost model offers valuable insights into the key determinants of endurance rowing performance, guiding the personalization of supplement strategies. Body weight emerged as the most influential predictor. This aligns with extensive research highlighting the critical role of body mass and composition in athletic success^75^, as they directly impact factors like power-to-weight ratio and energy availability^76^. The high ranking of average vertical jump height before exercise, an indicator of explosive power, also proved significant. This finding is consistent with studies demonstrating a relationship between vertical jump capabilities and rowing performance, suggesting that anaerobic power contributes to overall endurance capacity in rowers^77^. The importance of such power metrics is further supported by research on athletic development and training^78^.

Nutritional variables, including Previous meal time and the intake rates of CHO and PRO, were also identified as top-tier predictors. The significance of Previous meal time underscores the established principle of nutrient timing to optimize energy stores and physiological readiness for endurance activities^1,79,80^. The prominence of CHO and PRO intake rates directly reflects their fundamental roles in energy provision and muscle metabolism during sustained exercise^81–83^. The model’s sensitivity to these dietary inputs validates their central role in the personalized supplementation framework developed in this study. Notably, the increased prominence of these specific nutritional factors in the final model, compared to initial feature assessments on non-augmented data, may suggest that data augmentation allowed the model to better discern the nuanced impacts of these variables on performance.

The WGAN-GP augmented XGBoost model provides the foundation for the personalized supplement recommendation framework. Its achieved R^2^ value of 0.53 explains a notable portion of rowing performance variance, enabling more individualized guidance compared to generic advice. This study thereby establishes a viable data-driven methodology for advancing personalization in sports nutrition. However, the substantial unexplained variance necessitates cautious application. Model-generated recommendations should be interpreted as probabilistic guidance rather than definitive prescriptions, pending further validation.

The study develops and validates a predictive framework but does not include a real-world test of its recommendations. This represents a crucial distinction between a model’s predictive accuracy and its practical efficacy. Furthermore, the model is built upon a dataset with specific limitations. While strategies were employed to mitigate overfitting, the modest sample size means this risk remains a potential concern. The exclusive reliance on male participants is another key limitation, restricting the generalizability of the findings to female athletes. These limitations define a clear path for future research. The foremost priority is to conduct prospective intervention studies to validate the real-world efficacy and safety of the personalized recommendations. Such studies should also incorporate female and mixed-gender cohorts, and further model refinement will depend on integrating richer data, such as longitudinal athlete monitoring.

## Conclusion

This investigation aimed to develop and assess a ML system for personalized CPS to improve endurance rowing performance. Data were drawn from male with endurance rowing experience. These data included comprehensive baseline indicators, PRO intake rates, and rowing distance as the performance metric. A structured ML pipeline was implemented. This involved systematic feature selection, data augmentation, and the development of several regression models such as XGBoost, SVR, and MLP. These models predicted individual performance responses to varied CPS dosages, enabling tailored recommendations.

The XGBoost model, trained with WGAN-GP augmented data, was identified as the most effective overall predictor of rowing performance, delivering a strong combination of high predictive accuracy and superior model stability. This approach identified key predictors. These included body weight, explosive power, and nutritional inputs such as supplement intake rates and meal timing. The findings confirm that an integrated ML strategy can effectively predict endurance performance using individual athlete data. This data-driven methodology provides a robust foundation for developing personalized nutritional support in sports. Future research should prioritize prospective validation studies to assess the real-world impact of these personalized recommendations.

## Supplementary Information

Below is the link to the electronic supplementary material.

Supplementary Material 1

## Data Availability

The raw data for this study are not publicly available due to their use in ongoing research. However, the full code pipeline used for data processing, feature selection, data augmentation, model training, and analysis is publicly available in a GitHub repository. The repository includes numbered Jupyter Notebooks, a detailed README file with step-by-step instructions, and an environment configuration file for full reproducibility. The code can be accessed at: https://github.com/Michael1006-dev/personalized-nutrition-rowing.
