# Supplementary material for: Data-augmented machine learning for personalized carbohydrate-protein supplement recommendation for endurance
Source: Sci Rep. 2025 Nov 17;15:40181. doi: 10.1038/s41598-025-23989-7 (PMC12624012; doi:10.1038/s41598-025-23989-7)
Supplement: Supplementary file 1 — Supplementary Material 1 [file 41598_2025_23989_MOESM1_ESM.docx]

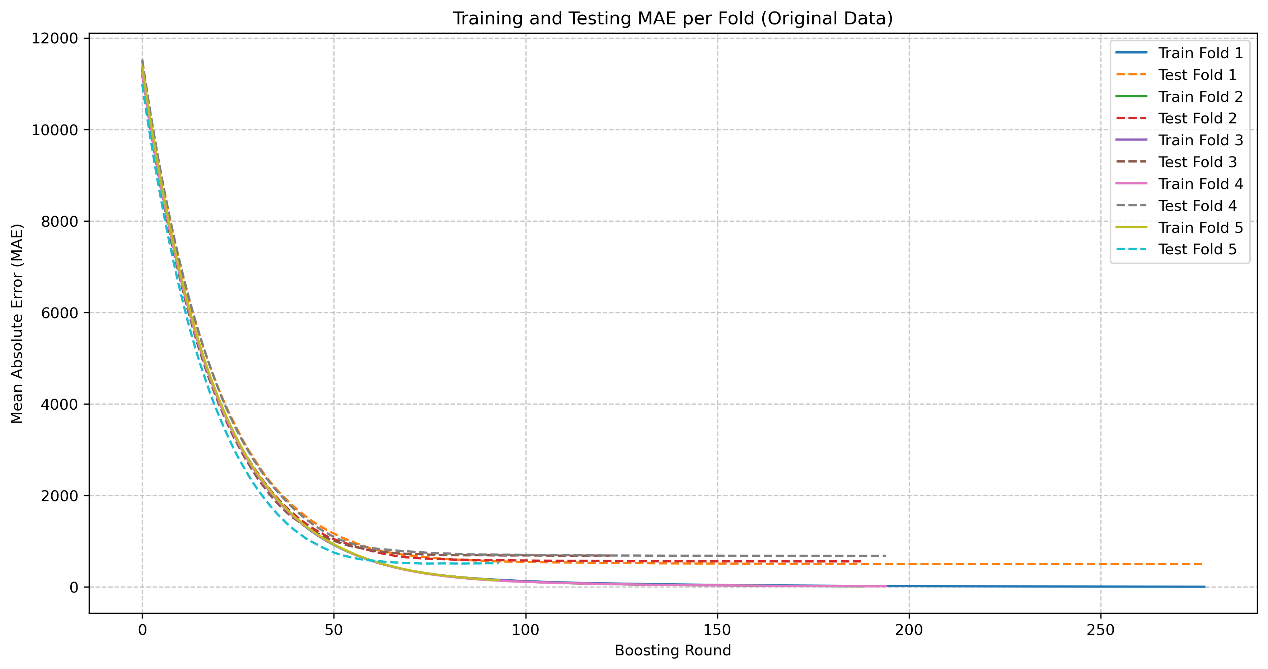


**Figure S1:** Learning dynamics of the XGBoost model during cross-validation on the original dataset.


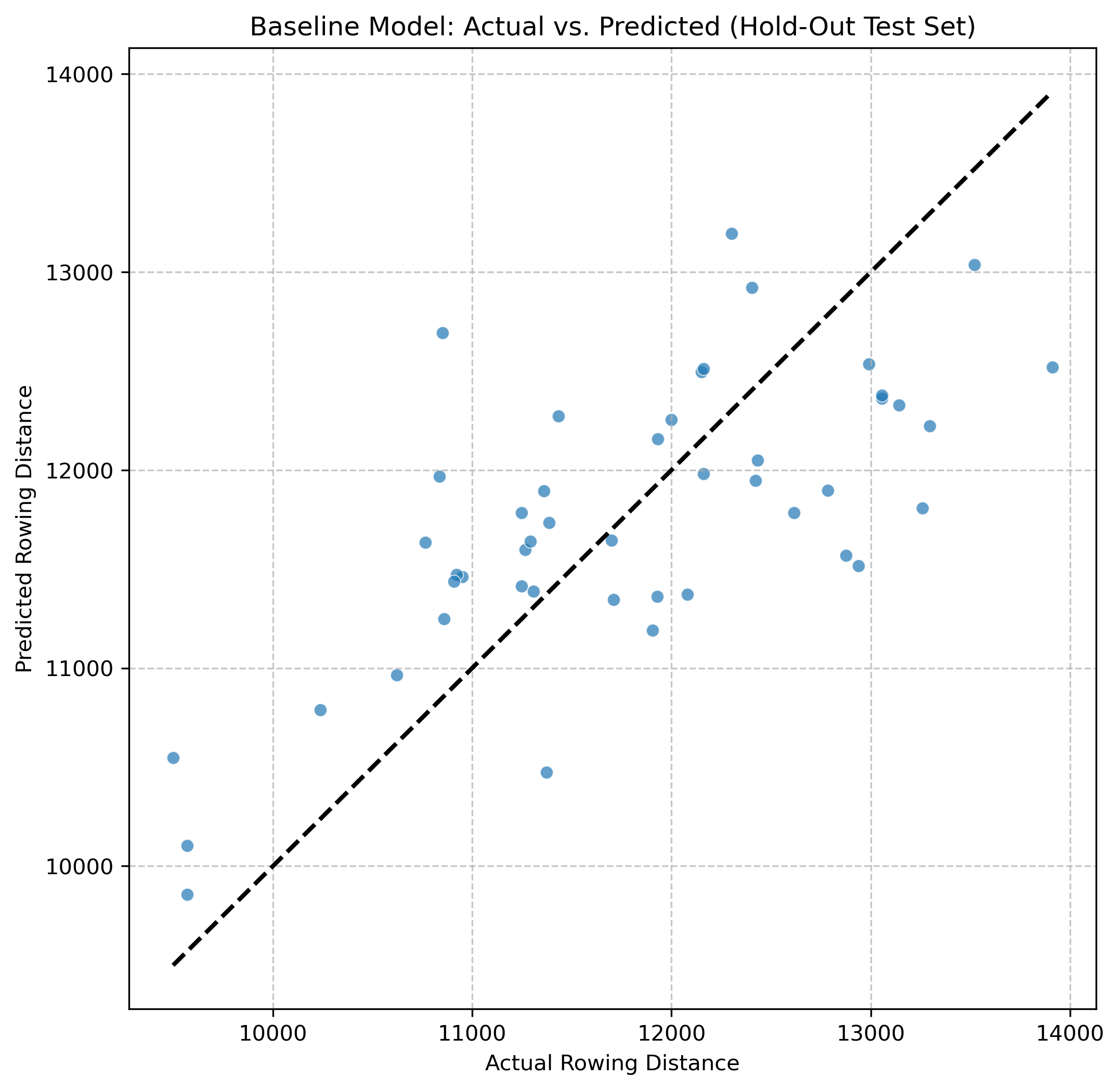


**Figure S2** Correlation between actual and predicted rowing distance for the baseline XGBoost model on the test set.


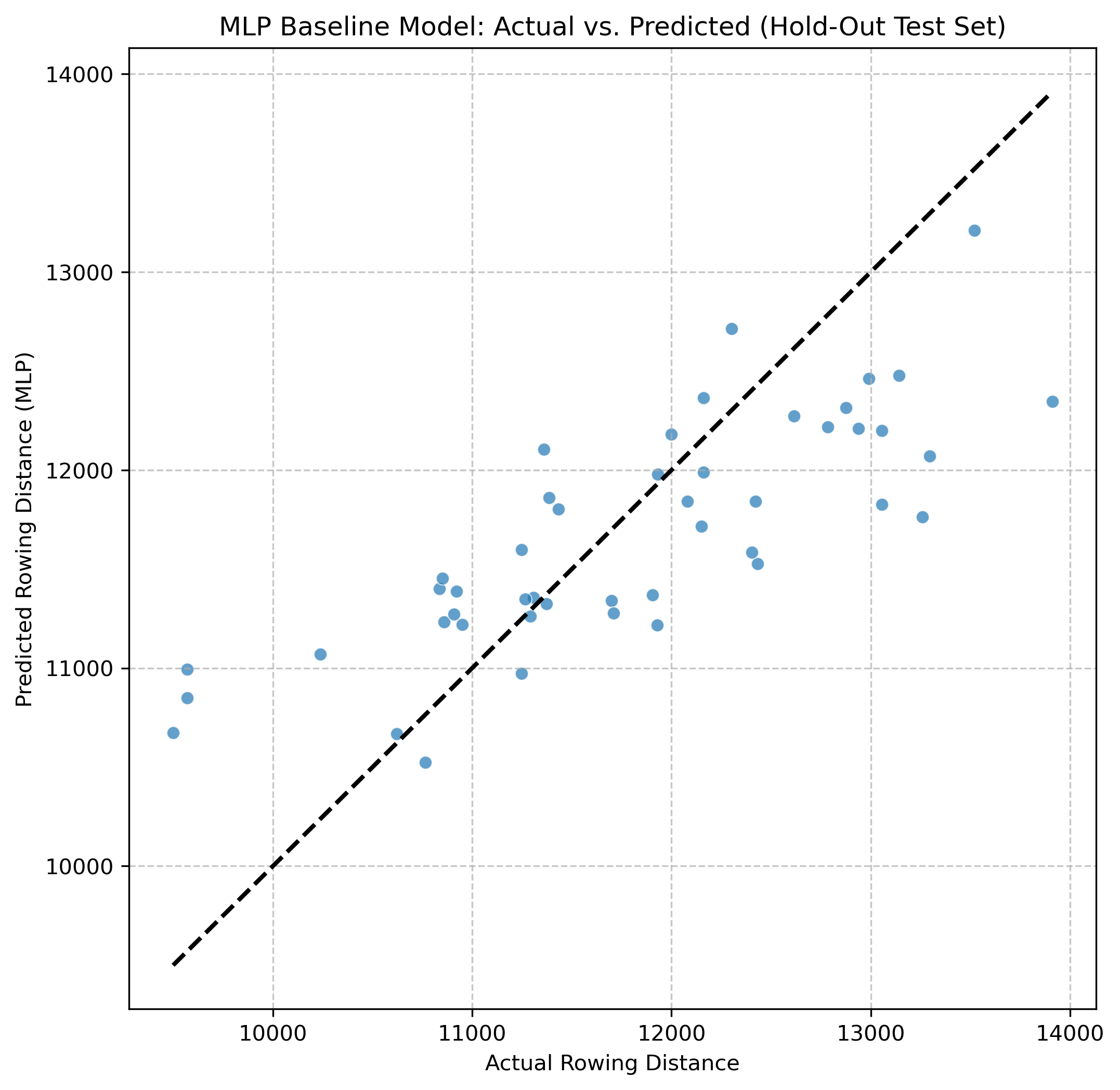


**Figure S3** Correlation between actual and predicted rowing distance for the baseline MLP model on the test set.


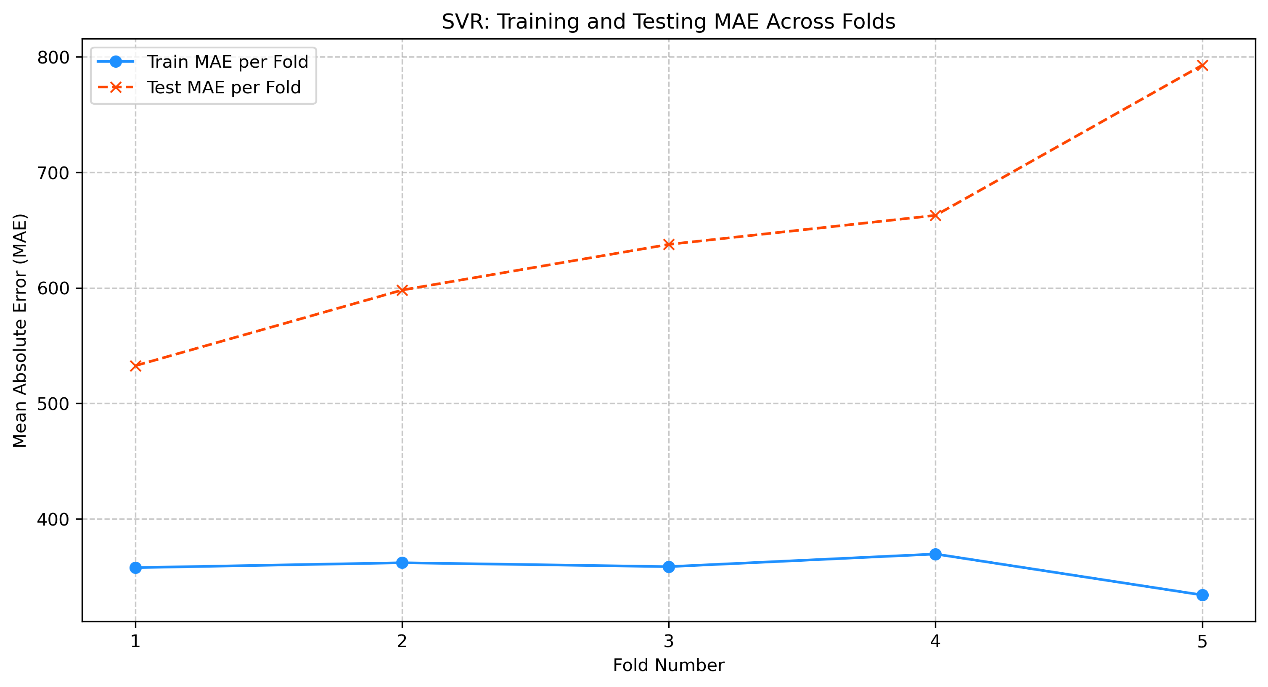


**Figure S4:** Learning dynamics of the SVR model during cross-validation on the original dataset.


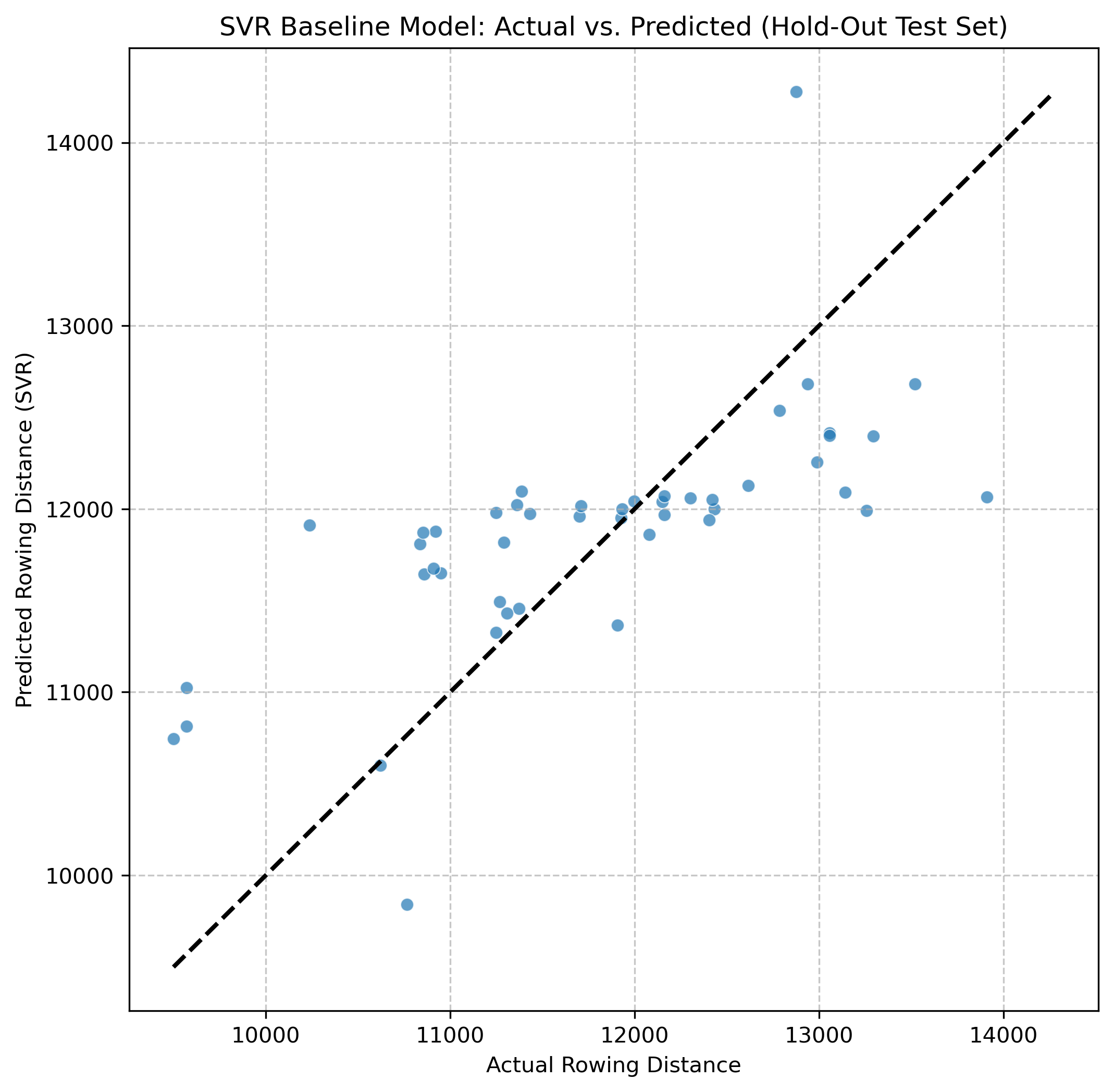


**Figure S5** Learning dynamics of the SVR model during cross-validation on the original dataset.


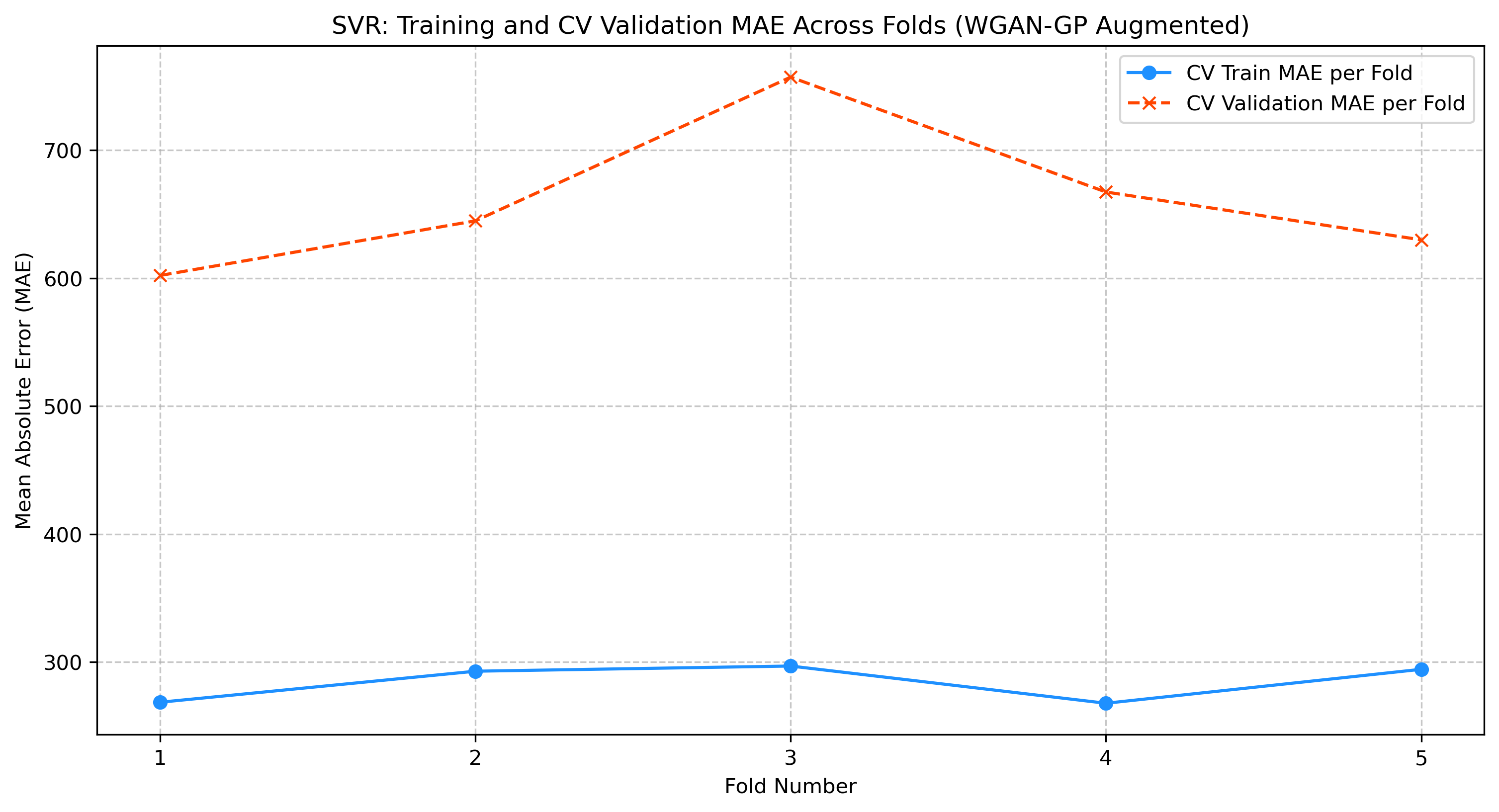


**Figure S6:** Learning dynamics of the SVR model during cross-validation on the WGAN-GP augmented dataset.


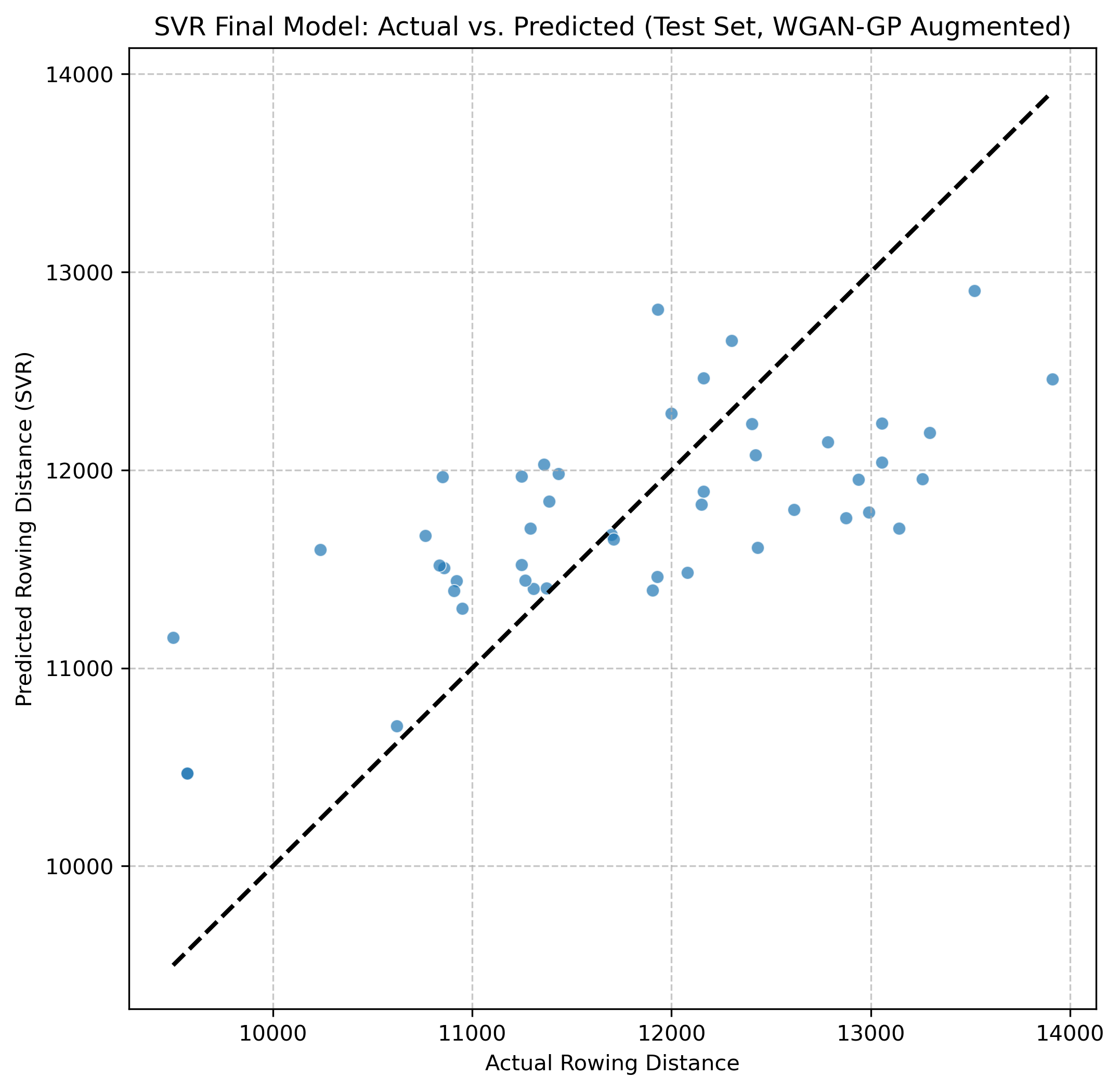


**Figure S7:** Correlation between actual and predicted rowing distance for the SVR model (trained with augmentation) on the test set.


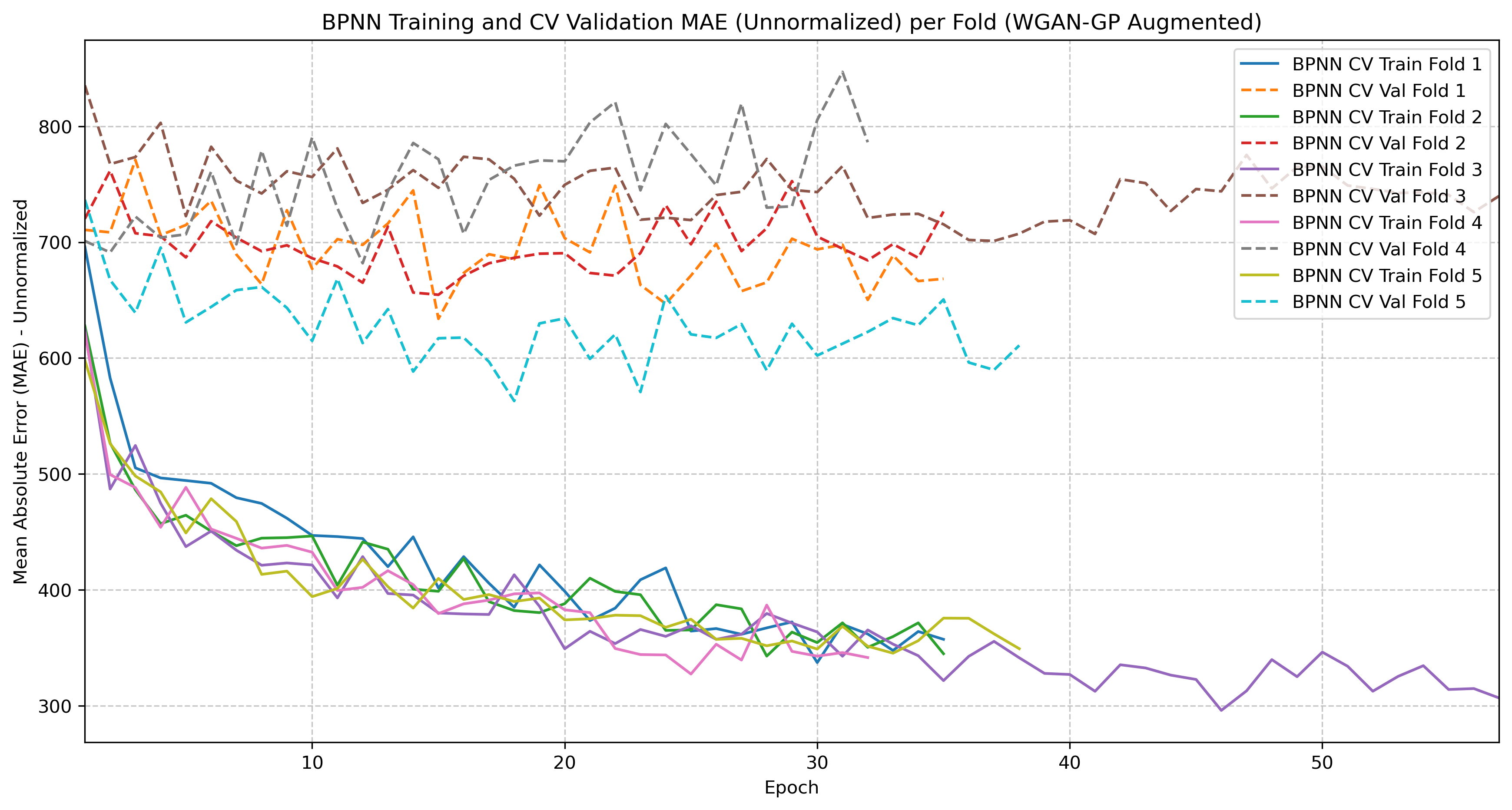


**Figure S8:** Learning dynamics of the MLP model during cross-validation on the WGAN-GP augmented dataset.


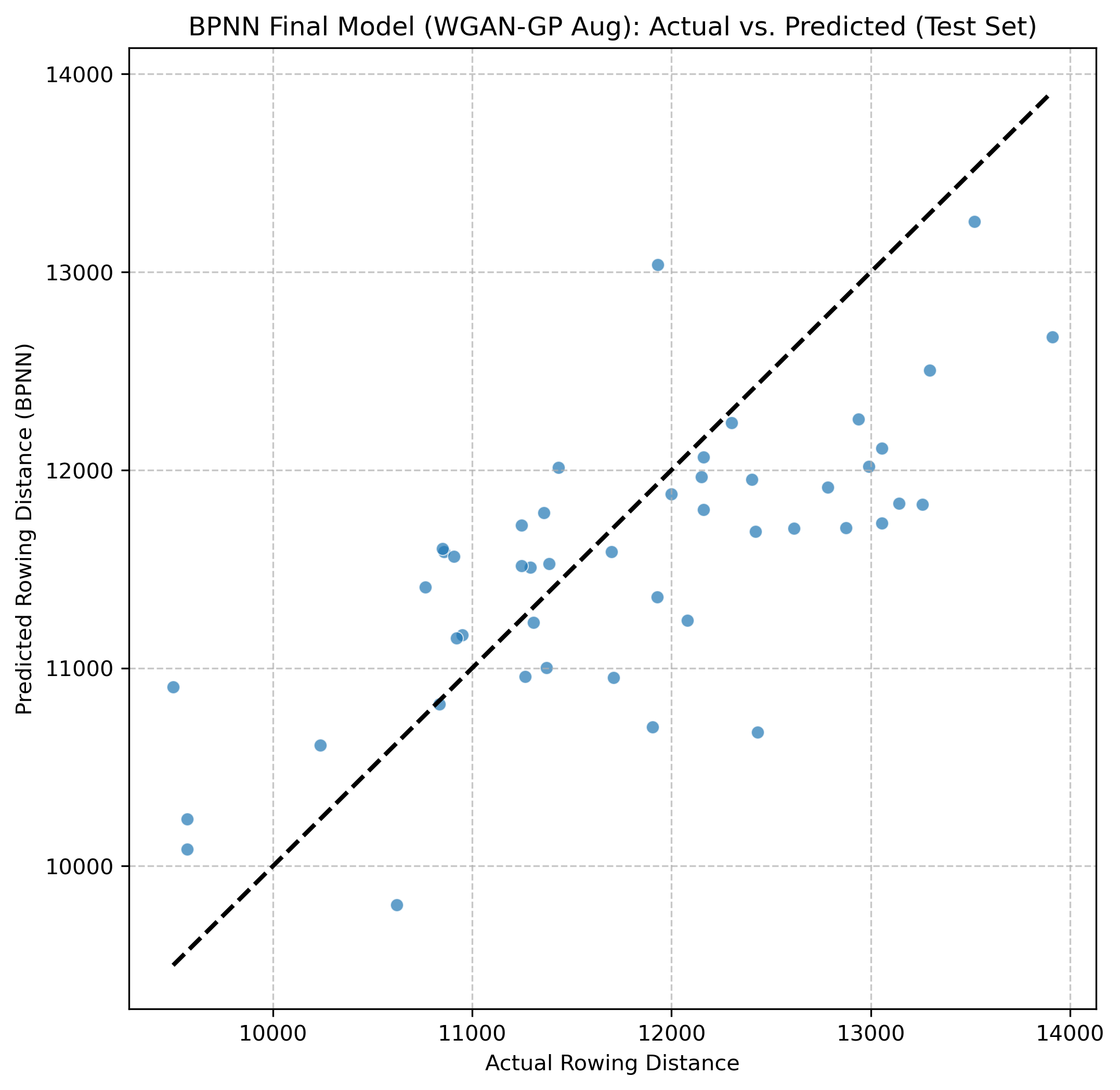


**Figure S9:** Correlation between actual and predicted rowing distance for the MLP model (trained with augmentation) on the test set.
